# Supplementary material for: Selling one’s future: over-indebtedness and the risk of poor mental health and the role of precarious employment – results from the Scania Public Health Cohort, Sweden
Source: BMJ Open. 2022 Nov 22;12(11):e061797. doi: 10.1136/bmjopen-2022-061797 (PMC9685259; doi:10.1136/bmjopen-2022-061797)
Supplement: Author's manuscript [file bmjopen-2022-061797.draft_revisions.pdf]

BMJ Open

BMJ Open is committed to open peer review. As part of this commitment we make the peer review history of every article we publish publicly available.

When an article is published we post the peer reviewers' comments and the authors' responses online. We also post the versions of the paper that were used during peer review. These are the versions that the peer review comments apply to.

The versions of the paper that follow are the versions that were submitted during the peer review process. They are not the versions of record or the final published versions. They should not be cited or distributed as the published version of this manuscript.

BMJ Open is an open access journal and the full, final, typeset and author-corrected version of record of the manuscript is available on our site with no access controls, subscription charges or pay-per-view fees (http://bmjopen.bmj.com).

If you have any questions on BMJ Open's open peer review process please email info.bmjopen@bmj.com

Selling one's future: Over-indebtedness and the risk of poor mental health and the role of precarious employment – results from the Scania Public Health Cohort, Sweden

Journal:	*BMJ Open*
Manuscript ID	bmjopen-2022-061797
Article Type:	Original research
Date Submitted by the Author:	07-Feb-2022
Complete List of Authors:	Östergren, Per-Olof; Lund University, Department of Clinical Sciences Malmö<br>Bodin, Theo; Karolinska Institutet; Center for Occupational and Environmental Medicine<br>Canivet, Catarina ; Lund University, Dept of Clinical Sciences Malmo<br>Moghaddassi, Mahnaz; Lund University, Department of Clinical Sciences Malmö<br>Vilhelmsson, Andreas; Lund University, Department of Laboratory Medicine
Keywords:	MENTAL HEALTH, EPIDEMIOLOGY, Health policy < HEALTH SERVICES ADMINISTRATION & MANAGEMENT, PUBLIC HEALTH

SCHOLARONE™
Manuscripts

I, the Submitting Author has the right to grant and does grant on behalf of all authors of the Work (as defined in the below author licence), an exclusive licence and/or a non-exclusive licence for contributions from authors who are: i) UK Crown employees; ii) where BMJ has agreed a CC-BY licence shall apply, and/or iii) in accordance with the terms applicable for US Federal Government officers or employees acting as part of their official duties; on a worldwide, perpetual, irrevocable, royalty-free basis to BMJ Publishing Group Ltd ("BMJ") its licensees and where the relevant Journal is co-owned by BMJ to the co-owners of the Journal, to publish the Work in this journal and any other BMJ products and to exploit all rights, as set out in our licence.

The Submitting Author accepts and understands that any supply made under these terms is made by BMJ to the Submitting Author unless you are acting as an employee on behalf of your employer or a postgraduate student of an affiliated institution which is paying any applicable article publishing charge ("APC") for Open Access articles. Where the Submitting Author wishes to make the Work available on an Open Access basis (and intends to pay the relevant APC), the terms of reuse of such Open Access shall be governed by a Creative Commons licence – details of these licences and which Creative Commons licence will apply to this Work are set out in our licence referred to above.

Other than as permitted in any relevant BMJ Author's Self Archiving Policies, I confirm this Work has not been accepted for publication elsewhere, is not being considered for publication elsewhere and does not duplicate material already published. I confirm all authors consent to publication of this Work and authorise the granting of this licence.

For peer review only

Selling one's future: Over-indebtedness and the risk of poor mental health and the role of precarious employment – results from the Scania Public Health Cohort, Sweden

Per-Olof Östergren[1], Theo Bodin[2,3], Catarina Canivet[1], Mahnaz Moghaddassi[1], Andreas Vilhelmsson[4]

1 Social Medicine and Global Health, Department of Clinical Sciences Malmö, Lund University, Lund, Sweden

2 Unit of Occupational Medicine, Institute of Environmental Medicine, Karolinska Institutet, Stockholm, Sweden

3 Center for Occupational and Environmental Medicine, Stockholm Region, Stockholm, Sweden

4 Division of Occupational and Environmental Medicine, Department of Laboratory Medicine, Lund University, Lund, Sweden

Corresponding author: Per-Olof Östergren, per-olof.ostergren@med.lu.se, +46 705160092

Word count abstract: 298

Word count article: 3885

For peer review only

Abstract

OBJECTIVES: The credit market has expanded rapidly, increasing the risk of over- indebtedness among those who lack secure employment or adequate income, an issue of concern in the Covid-19 aftermath.: We investigated the role of over-indebtedness for developing poor mental health, and whether this impact is modified by age, gender, educational level, or being in precarious employment.

METHODS: This is a cohort study where data was obtained from the Swedish Scania Public Health Cohort based on individuals randomly selected from the general adult population in Scania, southern Sweden, initiated in 1999/2000 with follow-ups in 2005 and 2010. Over-indebtedness was assessed by combining information on cash margin and difficulty in paying household bills. Mental health was assessed by General Health Questionnaire-12. Individuals with complete data on main variables were included, individuals with poor mental health at baseline were excluded, resulting in 1256 men and 1539 women.

RESULTS: Over-indebtedness was more common among women, among persons with a low educational level, born abroad, and with a precarious employment at baseline. The age-adjusted incidence rate ratio (IRR) for poor mental health in 2010 among individuals exposed to over-indebtedness in 1999/2000 or 2005 was 2.2 (95% CI: 1.7–2.8). Adjusting for educational level, country of origin, and precarious employment in 1999/2000 or 2005, yielded an IRR of 2.0; 95% CI: 1.6–2.6. A three-way interaction analysis indicated that a high level of education may act synergistically with over-indebtedness, regarding poor mental health among men.

CONCLUSIONS: Over-indebtedness was related to unfavourable societal power relations, i.e. social class, gender, and foreign birth. Precarious employment was independently linked to poor mental health, especially among men, and may also mediate the effect by over-indebtedness. The Covid-19 pandemic might entail increased over-indebtedness, which should be acknowledged in policies aiming at buffering the social effects of the pandemic.

For peer review only - http://bmjopen.bmj.com/site/about/guidelines.xhtml

Strengths and limitations of this study

- This was a prospective cohort study with 2795 vocationally active men and women randomly selected from the general adult population.
- The exposure and outcome variables have been used and validated in previous studies.
- Detailed information concerning employment history and details regarding current empolyment for each individual was used for determining precarious empolyment status.
- Non-response and missing data among respondents may have caused some selection bias.
- We lacked access to an objective measure regarding over-indebtedness, which may have caused misclassification.

For peer review only

For peer review only - http://bmjopen.bmj.com/site/about/guidelines.xhtml

Introduction

Credit plays a paramount role in modern economy, since it increases the possibility to make investments that can generate future financial and other gains and to allocate lifetime income in a more optimal relation to need. In a health perspective, such resources are very likely related to opportunities for improved health over the life-course. However, when the credit market targets those who cannot make ends meet, this can cause increasing indebtedness, which can lead to poorer health, both due to loss of material and other resources and to exposure to growing stress and worries about one´s loss of control over the household economy. Thus, the proportion of individuals who are prone to over-indebtedness will most likely expand considerably in the aftermath of the covid-19 pandemic. Moreover, indebtedness could be expected to have a differential effect on health, much depending on factors such as socioeconomic position, country of birth (i.e. migration background), and age.

The credit market has expanded over the last decades leading to a build-up of consumer debt. However, it was not until the financial crisis in 2007-2008 that an increased proportion of households that had difficulties making ends meet was noted in the EU.[1] As of 2016, the household debt in relation to disposable income was more than twice as high in the OECD compared to 1996.[2] Measured as share of GDP, the indebtedness of Swedish households has doubled since the 1990s.[3] According to public reports, the number of overindebted individuals in Sweden amounts to between 6 and 18 per cent of the population, depending on definition. The subsequent yearly societal cost is estimated to 30 billion SEK.[4] The credit market has also changed in terms of type of credit providers and types of 'products', e.g. so called 'pay-day loans' as a temporary financial solution when financial means are insufficient to cover daily cost of living because of inadequate income and lack of savings.[5] These types of loans have at the same time become very accessible via the Internet and mobile phones. Since the interest rates and fees generally are very high for these loans, this could

For peer review only - http://bmjopen.bmj.com/site/about/guidelines.xhtml

quickly lead to a spiral of indebtedness where the loan-taker in practice ends up owing the lender a substantial proportion of his/her future income.[6]

When the cost of accumulated credit implies an inability to meet recurring expenses and thus results in over-indebtedness, this situation could represent an emerging significant stressor and determinant of ill health. This source of health inequality, based on financial mechanisms, is likely to be overlooked in research on income or wealth and health, since information concerning over-indebtedness can not easily be retrieved from registers on income, wealth, or social transfers.

A growing number of scientific studies on the indirect and direct association between over-indebtedness and health have been published in the scientific literature. Systematic reviews have provided evidence that the financial recession is associated with an increase in suicide rates in high-income countries such as Europe and North America.[7] Further, socioeconomic mediators such as unemployment, income decline, and unmanageable debts are significantly associated with poor mental wellbeing, increased rates of common mental disorders, substance-related disorders, and suicidal behaviours.[8-11] Individuals who cannot make their loan payments have been found to suffer from depression and have suicidal ideation more often compared to those without such problems.[12] Another study investigating predictors of mental health in people on the verge of bankruptcy found that perceived financial strain was an important predictor of poor mental health.[13]

High debt relative to available assets has also been associated with poorer self-assessed general health, higher diastolic blood pressure, obesity, poorer health-related behaviour, and myocardial infarction.[14] The effect of over-indebtedness on health has been found to be stronger for women than for men.[15] In a life-course perspective, poor self-rated health has been found to be even more prevalent among those who have experienced economic stress both early in life and later on.[16]

For peer review only - http://bmjopen.bmj.com/site/about/guidelines.xhtml

According to a systematic review and meta-analysis examining the relationship between personal debt and mental health,[17] ten epidemiological studies with nationally representative samples of the general population were found, whereof seven came from the UK and nine out of ten were cross-sectional. Unsurprisingly, very high odds ratios for different mental health outcomes were found. ¨In many cases the causality pattern could be described as a vicious circle, which could start either by a decrease in income or in health. Poor mental health provoked by over-indebtedness tends to increase the risk of further financial problems and so forth.[18] Moreover, over-indebtedness or coping with excessive credit is associated with subjective distress such as shame, anxiety, and apathy.[19]

To the best of our knowledge, evidence regarding the role of precarious employment as a mediator of modifier of the impact of over-indebtedness on mental health is lacking.

In summary, previous research relies heavily on cross-sectional data from the UK, why studies from other national contexts are needed. Moreover, because of the plausible bidirectional causal pathways, prospective studies of long duration with several assessments of both types of variables, over-indebtedness as well as mental health, are preferable. Also, as mentioned above, little is known about the role of precarious employment and the differential health effects across sociodemographic groups.

Objectives

The aim of the study was to investigate the role of over-indebtedness for the development of poor mental health in a longitudinal perspective, and whether this impact is modified by age, gender, educational level, housing tenure, or being in a precarious employment situation.

Methods

For peer review only

Study population

The present cohort was established in 1999/2000 and followed up in 2005 and 2010.[20] At baseline, a postal questionnaire was sent out to 25 000 men and women, 18–80 years old. These individuals were randomly selected from the population register, such that equal representation was achieved from all municipalities in the county of Scania (population 1.3 million), Sweden. In total, 13589 out of 23437 eligible individuals returned a completed questionnaire (response rate 58%). All of those who responded at baseline and still residing in the county were invited to follow-up after five and ten years. Out of 12002 respondents alive and still living in the region after 10 years, there were 8206, i.e. 68%, who also participated in the 2005 and 2010 inquiries.

A comparison between these 8206 respondents and the corresponding Scania general population at baseline [20] showed that younger individuals, men, and individuals with a low educational level were slightly underrepresented among respondents. However, a recently published study from this cohort, showed very little proof of selection bias regarding common health outcomes, includung mental health.[21]

Since one aim of the study was to look into the relationship between precarious employment and over-indebtedness and mental health, we firstly excluded individuals who were not vocationally active, i.e. at baseline were retired, on disability pension, or on long-term sick leave, and also those who answered that they did not wish to work a year from now (N=1744). Out of the remaining 6462 persons, those with lacking data on employment precariousness (N=2479) were excluded; out of these, 1106 were 55–80 years old at baseline, i.e. were qualified for old-age retirement during the studied window of time. Further, we excluded individuals who lacked baseline data regarding the variables included in the multivariable analyses (rather than assigning them as ‘internal’ missing); i.e. housing tenure (N=159) or educational level (N=158), also those lacking baseline and 2005 information on

For peer review only - http://bmjopen.bmj.com/site/about/guidelines.xhtml

over-indebtedness (N=249), furthermore those who lacked information regarding mental health at all follow-ups (N=161). Lastly, in order to reduce the influence of reverse causation, we also excluded individuals with poor mental health at baseline (N=1563), instead of controlling for this in the multivariable analysis. Thus, the final study population consisted of 2795 individuals, 1256 men and 1539 women.

Outcome variable – poor mental health

Mental health was measured at all three time points of assessment, using the 12-item version of the General Health Questionnaire (GHQ-12). We used the 0-0-1-1 scoring method (range 0–12) recommended by the creators of the instrument, with poor mental health or 'GHQ-caseness' defined as a score of 2 or higher.[22, 23]

Over-indebtedness

Two items have been used previously in several Swedish studies to evaluate economic hardship, namely inability to meet expenses and lacking cash reserves.[24] For the former we used the question 'How often during the last 12 months have you had problems paying your bills?' and for the latter, 'In case of an unforeseen emergency, do you have the capacity to raise 12000 SEK *(approximately 1100 EUR)* in a week's time?' This amount was raised to 14000 SEK, approximately 1300 EUR, in the 2010 survey. Over-indebtedness was defined as affirming 'about half of the months' (or more often) on the first question and answering 'no' on the second.

Other variables

Age was classified into four groups, except in the interaction analysis, where it was dichotomised, and in the multivariate analysis, where it was used as a continuous variable. Country of origin was

For peer review only

recorded as ‘born in Sweden’ (yes/no). Educational level at baseline was determined by the self-reported total years of formal education and classified into four groups. Housing tenure was dichotomised as either owned or rented residence. For employment precariousness, we used a dichotomous variable, based on a combination of data on present unemployment, episode of involuntary unemployment during the past three years (no/yes), currently temporarily vs. permanently employed, and perceived job insecurity.[25, 26]

Statistical methods

The relationships between background factors and poor mental health in 2010 are presented as percentages and age-adjusted incidence rate ratios (IRR), which are a good estimate of relative risks, using a modified Poisson regression model with robust standard errors.[27] In the multivariate analysis, over-indebtedness was tested against the outcome with the stepwise addition of educational level, country of origin, and precarious employment. The tests for effect modification were performed with simple dummy variables. The synergy indices were calculated as proposed by Rothman.[28] according to which a synergy index > 1 indicates a synergistic effect, and a synergy index < 1 an antagonistic effect between two exposure variables. In the interaction analysis, educational level was dichotomised as ’12 years or less’ versus ’13 years or more’. There was a negative relationship between educational level and over-indebtedness (see Results), while the relationship with the outcome variable poor mental health at follow-up was positive (see Results), and therefore a high educational level was designated as exposure.

Two standard statistical analysis programmes were used, IBM SPSS Statistics for Windows, Version 22.0, Armonk, NY: IBM Corp. and Stata Statistical Software: Release 12, College Station, TX: StataCorp LP.

For peer review only

Patient and public involvement statement

Patient or public involvement in the design, reporting or dissemination of results was not possible.

Results

Table 1 shows the prevalence of over-indebtedness in the total study population at baseline and also separately by gender. Overall, the prevalence of overindebted individuals was 4.2%, but with a clear difference between men and women (2.8 versus 5.3%, respectively). The only clear age-related difference in over-indebtedness consisted of a low prevalence in participants aged 55 and above. Over-indebtedness was more common in those with an educational level of 10–12 years, vs. those with less or with more years. When educational level was dichotomised as '12 years or less' vs. '13 years or more', 5.5% of those with a low educational level were overindebted at baseline, vs. 2.7% of those with a high educational level (data not shown in tables). Over-indebtedness was three times as common among those born in another country than Sweden. As perhaps expected, over-indebtedness was almost four times as prevalent among those who reported a precarious employment situation and among those who rented their dwelling (compared to those who owned it). The gender pattern was largely similar for all factors.

For peer review only

Table 1

Background characteristics at baseline in 1999/2000 of 1256 men and 1539 women from the Scania Public Health Cohort, in relation to over-indebtedness.

		All				Men				Women			
		Total	No	Yes[a]	% Yes	Total	No	Yes	% Yes	Total	No	Yes	% Yes
Total		2795	2679	116	4.2	1256	1221	35	2.8	1539	1458	81	5.3
Age	18–24	207	196	11	5.3	96	92	4	4.2	111	104	7	6.3
	25–34	621	596	25	4.0	278	272	6	2.2	343	324	19	5.5
	35–44	750	710	40	5.3	324	314	10	3.1	426	396	30	7.0
	45–54	966	930	36	3.7	430	416	14	3.3	536	514	22	4.1
	55–63	251	247	4	1.6	128	127	1	0.8	123	120	3	2.4
Educational level	9 y or less	502	484	18	3.6	265	256	9	3.4	237	228	9	3.8
	10–12	954	892	62	6.5	437	416	21	4.8	517	476	41	7.9
	13–14	633	609	24	3.8	253	250	3	1.2	380	359	21	5.5
	15 y or more	706	694	12	1.7	301	299	2	0.7	405	395	10	2.5
Born in Sweden	Yes	2588	2494	94	3.6	1163	1136	27	2.3	1425	1358	67	4.7
	No	207	185	22	10.6	93	85	8	8.6	114	100	14	12.3
Precarious employment	No	2051	2004	47	2.3	943	926	17	1.8	1108	1078	30	2.7
	Yes	744	675	69	9.3	313	295	18	5.8	431	380	51	11.8
Housing tenure	Owned	2176	2112	64	2.9	990	969	21	2.1	1186	1143	43	3.6
	Rented	619	567	52	8.4	266	252	14	5.3	353	315	38	10.8

[a] Over-indebted at baseline

For peer review only

Table 2 shows the results of the longitudinal analysis, where poor mental health at follow-up in 2010 was used as the outcome. The age-adjusted risk for poor mental health at follow-up in overindebted individuals, measured as incidence rate ratio (IRR), was 2.2 (95% CI: 1.7–2.8) in the entire group, 2.6 (1.8–3.9) for men, and 1.9 (1.4–2.6) for women. The risk for poor mental health was four times higher in those who were 18–24 years at baseline, with an IRR of 4.1 (2.3–7.4), and decreased successively with increasing age, compared to those who were 55 or older at baseline, which was used as the reference group. That pattern was similar in men and women. Those with an educational level of 13–14 years at baseline had a slightly higher risk of poor mental health at follow-up (IRR=1.4; 95% CI: 1.01–2.0) than those with 9 years or less, which was used as the reference. When educational level was dichotomised, 15.1% of those with a high educational level had poor mental health at follow-up vs. 13.9% of those with a low educational level (data not shown in tables). Foreign birth was related to poor mental health in men only, with an IRR of 1.6 (1.01–2.6). Having a precarious employment situation during the period 1999/2000 to 2005 was statistically significantly related to poor mental health in 2010 (IRR= 1.4; 95% CI:1.2–1.7), albeit also here with a gender difference; the IRR for men was 1.8 (1.3–2.4) and for women 1.2 (0.95–1.5). Housing tenure (rented dwelling) at baseline was not related to poor mental health in 2010.

For peer review only

For peer review only - http://bmjopen.bmj.com/site/about/guidelines.xhtml

Table 2
Background characteristics from 1999/2000 through 2005 in relation to poor mental health in 2010.

		All				Men				Women			
		N cases poor mental health [a]	% cases poor mental health	IRR [b]	95% CI [c]	N cases poor mental health	% cases poor mental health	IRR	95% CI	N cases poor mental health	% cases poor mental health	IRR	95% CI
Total		404	14.5			144	11.5			260	16.9		
Over-indebtedness in 1999/2000 or 2005, at least once	No	348	13.3	1		124	10.4	1		224	15.7		1
	Yes	56	31.8	2.2	1.7–2.8	20	31.3	2.6	1.8–3.9	36	32.1	1.9	1.4–2.6
Age in 1999/2000	18–24	44	21.3	4.1	2.3–7.4	17	17.7	5.7	2.0–16.3	27	24.3	3.3	1.6–6.8
	25–34	125	20.1	3.9	2.2–6.8	46	16.5	5.3	2.0–14.4	79	23.0	3.1	1.6–6.1
	35–44	114	15.2	2.9	1.7–5.1	41	12.7	4.0	1.5–11.1	73	17.1	2.3	1.2–4.5
	45–54	108	11.2	2.2	1.2–3.8	36	8.4	2.7	0.97–7.4	72	13.4	1.8	0.9–3.6
	55–63	13	5.2	1		4	3.1	1		9	7.3	1	
Educational level in 1999/2000	9 y or less	47	9.4	1		18	6.8	1		29	12.2	1	
	10–12	155	16.2	1.3	0.9–1.8	61	14.0	1.4	0.8–2.5	94	18.2	1.1	0.8–1.7
	13–14	107	16.9	1.4	1.01–2.0	27	10.7	1.2	0.7–2.1	80	21.1	1.4	0.9–2.1
	15 y or more	95	13.5	1.2	0.8–1.6	38	12.6	1.4	0.8–2.5	57	14.1	0.9	0.6–1.4
Born in Sweden	Yes	369	14.3	1		128	11.0	1		241	16.9	1	
	No	35	16.9	1.2	0.9–1.6	16	17.2	1.6	1.01–2.6	19	16.7	1.0	0.6–1.5
Precarious employment in 1999/2000 or 2005, at least once	No	211	12.0	1		69	8.5	1		142	15.0	1	
	Yes	193	18.6	1.4	1.2–1.7	75	16.7	1.8	1.3–2.4	118	20.0	1.2	0.95–1.5
Housing tenure in 1999/2000	Owned	292	13.4	1		101	10.2	1		191	16.1	1	
	Rented	112	18.1	1.1	0.9–1.4	43	16.2	1.3	0.9–1.9	69	19.5	1.0	0.8–1.3

[a] Poor mental health is defined as GHQ-caseness; [b] IRR, incidence rate ratio, age-adjusted; [c] CI, confidence interval

For peer review only

For peer review only - http://bmjopen.bmj.com/site/about/guidelines.xhtml

Table 3 shows the effect on the risk estimate for poor mental health at follow-up when exposed to over-indebtedness during the period 1999/2000 to 2005, with a step-wise introduction of the main covariates. In the final step, when precarious employment situation during 1999/2000 to 2005 was introduced into the analysis, the risk estimate for poor mental health regarding exposure to over-indebtedness 1999/2000 to 2005 decreased about 10% for the entire group. The decrease in IRR in the fully adjusted model was slightly larger for men than for women. As a general remark, we find it very likely that this is a case of over-adjustment, since all the introduced covariates could be included in the same causal chains, as well as in separate ones, and we have no way to disentangle this.

For peer review only

Table 3

Over-indebtedness at least once in 1999/2000 or 2005 in relation to the outcome poor mental health at follow-up in 2010, with forward stepwise addition of potential confounders; educational level in 1999/2000, country of origin, and precarious employment at least once in 1999/2000 or 2005.

			Model 1 Age-adjusted		Model 2 Model 1 + adjusted for educational level in 1999/2000		Model 3 Model 2 + adjusted for country of origin		Model 4 Model 3 + adjusted for precarious employment in 1999/2000 or 2005	
			IRR[a]	95% CI[b]	IRR	95% CI	IRR	95% CI	IRR	95% CI
All	Over-indebtedness	Yes versus no	2.2	1.7–2.8	2.2	1.7–2.8	2.2	1.7–2.8	2.0	1.6–2.6
	Educational level	9 years or less			1		1		1	
		10–12 years			1.2	0.9–1.7	1.2	0.9–1.7	1.3	0.9–1.8
		13–14 years			1.4	1.03–2.0	1.4	1.03–2.0	1.5	1.1–2.1
		15 years or more			1.2	0.9–1.7	1.2	0.9–1.7	1.3	0.9–1.8
	Born in Sweden	No versus yes					1.1	0.8–1.4	1.0	0.7–1.4
	Precarious employment	Yes versus no							1.3	1.1–1.6
Men	Over-indebtedness	Yes versus no	2.6	1.8–3.9	2.7	1.8–4.2	2.6	1.7–4.0	2.2	1.4–3.5
	Educational level	9 years or less			1		1		1	
		10–12 years			1.4	0.8–2.4	1.4	0.8–2.4	1.4	0.8–2.4
		13–14 years			1.2	0.7–2.2	1.3	0.7–2.2	1.2	0.7–2.2
		15 years or more			1.6	0.9–2.8	1.6	0.9–2.7	1.6	0.9–2.8
	Born in Sweden	No versus yes					1.3	0.8–2.1	1.2	0.7–1.9
	Precarious employment	Yes versus no							1.6	1.2–2.3
Women	Over-indebtedness	Yes versus no	1.9	1.4–2.6	1.9	1.4–2.5	1.9	1.4–2.6	1.8	1.4–2.5
	Educational level	9 years or less			1		1		1	
		10–12 years			1.1	0.7–1.7	1.1	0.7–1.7	1.1	0.8–1.7
		13–14 years			1.4	0.9–2.1	1.4	0.9–2.1	1.4	0.9–2.1
		15 years or more			1.0	0.6–1.5	1.0	0.6–1.5	1.0	0.6–1.5
	Born in Sweden	No versus yes					0.9	0.6–1.4	0.9	0.6–1.3
	Precarious employment	Yes versus no							1.1	0.9–1.4

[a] IRR, incidence rate ratio; [b] CI, confidence interval

For peer review only - http://bmjopen.bmj.com/site/about/guidelines.xhtml

As it is of considerable interest to investigate whether there are certain segments of the population that are more vulnerable concerning the risk of developing poor mental health when exposed to over-indebtedness, we examined this concerning age and level of education. As seen in Table 4A, there was no indication of a synergistic effect of young age and over-indebtedness on the risk of poor mental health at follow-up. However, as seen in Table 4B, while there was likewise no interaction between a high educational level and over-indebtedness in women, a higher risk was seen in men with this particular combination of risk factors. The IRR for men was 3.8 (95% CI: 1.5–9.6), and the resulting synergy index 1.8.

For peer review only

Table 4A

Interaction analyses, by gender, with synergy indices regarding age group in 1999/2000 and over-indebtedness at least once in 1999/2000 or 2005, in relation to poor mental health in 2010.

	All					Men					Women				
Age group in 1999/2000 (18–34 vs 35–63) and over-indebtedness in 1999/2000 or 2005	N	% cases poor mental health	IRR[a]	95% CI[b]	SI[c]	N	% cases poor mental health	IRR[a]	95% CI[b]	SI[c]	N	% cases poor mental health	IRR[a]	95% CI[b]	SI[c]
Older & No over-indebtedness	1859	11.0	1			844	8.4	1			1015	13.2	1		
Older & Over-indebtedness	108	27.8	2.5	1.8–3.5		38	26.3	3.1	1.8–5.6		70	28.6	2.2	1.5–3.2	
Younger & No over-indebtedness	760	18.8	1.7	1.4–2.1		348	15.2	1.8	1.3–2.5		412	21.8	1.7	1.3–2.1	
Younger & Over-indebtedness	68	38.2	3.5	2.5–4.8	1.1	26	38.5	4.6	2.7–7.8	1.2	42	38.1	2.9	1.9–4.4	1.0
	2795					1256					1539				

[a] IRR, incidence rate ratio; [b] CI, confidence interval; [c] SI, synergy index

For peer review only - http://bmjopen.bmj.com/site/about/guidelines.xhtml

Table 4B

Interaction analyses, by gender, with synergy indices regarding educational level in 1999/2000 and over-indebtedness at least once in 1999/2000 or 2005, in relation to poor mental health in 2010.

	All					Men					Women				
Educational level in 1999/2000 and over-indebtedness in 1999/2000 or 2005	N	% cases poor mental health	IRR[a]	95% CI[b]	SI[c]	N	% cases poor mental health	IRR[a]	95% CI[b]	SI[c]	N	% cases poor mental health	IRR[a]	95% CI[b]	SI[c]
Low & No over-indebtedness	1336	12.4	1			649	9.9	1			687	14.8	1		
Low & Over-indebtedness	120	30.0	2.2	1.5–3.1		53	28.3	2.5	1.4–4.4		67	31.3	2.0	1.2–3.1	
High & No over-indebtedness	1283	14.2	1.1	0.9–1.4		543	11.0	1.1	0.8–1.5		740	16.5	1.1	0.8–1.4	
High & Over-indebtedness	56	35.7	2.6	1.6–4.1	1.2	11	45.5	3.8	1.5–9.6	1.8	45	33.3	2.1	1.2–3.6	1.0
	2795					1256					1539				

[a] IRR, age-adjusted incidence rate ratio; [b] CI, confidence interval; [c] SI, synergy index

For peer review only

Discussion

This study confirmed that vocationally active individuals who are exposed to over-indebtedness have an increased risk of developing poor mental health. Age-adjusted estimates showed that the risk was almost tripled for men and doubled for women in this general adult population sample of vocationally active individuals (18–65 years). Considering that about 4 % of all individuals in the cohort were exposed to over-indebtedness at baseline and probably substantially more during the follow-up period, over-indebtedness should be regarded as an important general population risk factor for poor mental health. This is pertinent especially in the aftermath of the Covid-19 pandemic, during which the number of vulnerable individuals in the adult population is likely to increase considerably.

At the same time, especially from a health equity perspective, it must be acknowledged that the prevalence and to some extent the impact of exposure to over-indebtedness in the population depend on sociodemographic circumstances. The reason that younger individuals are more exposed to over-indebtedness than middle-aged or older ones may partly have a 'life-course' explanation (mortgage loans are taken early in life and paid off over time), but there is also a generational effect due to the expansion of credit markets over time. For the other group disparities in over-indebtedness, reasons must be sought elsewhere. Women were more exposed than men, foreign-born more than those born in Sweden, and those with a low educational level more than those with a high level. In short, it seems that the risk for over-indebtedness follows all the main societal power structures, i.e. gender, social class, and ethnicity. Therefore, it is an important factor to consider when formulating and implementing policies for promoting health equity.

Regarding socioeconomic factors that logically should be strongly related to over-indebtedness, i.e. precarious employment and housing ownership (i.e. non-ownership), we found that both these factors were strongly related to over-indebtedness, but that only precarious employment was also related to the risk of developing poor mental health during our observation period. However, when we performed an analysis to control for confounding from

For peer review only - http://bmjopen.bmj.com/site/about/guidelines.xhtml

precarious employment, the impact of over-indebtedness on poor mental health was only moderately reduced. Considering a bidirectional relation between over-indebtedness and poor mental health, the choice to exclude individuals with poor mental health at baseline could have yielded an underestimation of the true association between the variables.

Health inequality could be the result of both differential exposure and differential impact when once exposed.[29] From the discussion above, it seems obvious that there is a clear case of differential exposure regarding over-indebtedness and sociodemographic factors. When we tested by means of two-way interactions whether we in addition could find any evidence for a differential impact, we concluded that this was not the case regarding age (Table 4A). We noted, however, that the combination of a high educational level and over-indebtedness (Table 4B) was associated with a synergy index of 1.0 in women, whereas the corresponding index in men was 1.8, thus indicating that these factors may act synergistically in a detrimental way for men. One could speculate that over-indebtedness may be especially hard to endure for a well-educated man, since over-indebtedness implies that one is financially not ‘in control’, whereas gender-based stereotypes of masculinity imply the opposite. Added to that might be the frustration caused by having invested many years in education and not being ‘rewarded’ by economic stability.

As shown in many studies as well as in this one, the basic power relations in society, among which would be those determined by gender, social class, and ethnicity, show a strong relation to the risk of becoming overindebted. This is not surprising, because all the above mentioned power relations determine access to factors that could avoid a short- or long-term situation where credit is needed for securing stable living conditions. Not being able to do so will induce both a loss of control and a loss of material resources, situations that have been shown to increase individuals’ susceptibility to poor health development, especially poor mental health. However, the ‘Faustian deal’ of using credit in this situation could make things worse, since a spiral of failing credit and declining mental health could place an individual in a situation where he or she in fact has sold a considerable part of future earnings to a moneylender institute. The possibility of having such debts cancelled in

For peer review only - http://bmjopen.bmj.com/site/about/guidelines.xhtml

a regulated way, which is open to business enterprises through the legal bankruptcy framework, is very restricted for individuals in the Swedish context. This is particularly problematic in a health equity perspective because of the strong association between societal power structures and the risk of becoming overindebted, all of which could be magnified in the wake of the covid-19 pandemic.

The results of this study are therefore very relevant for policy, not only regarding public health and health equity, but also for policy formulation and implementation in the judicial and social realms.

Strengths and weaknesses

This study utilises a prospective design using a large randomly selected general population sample of individuals in the occupationally active age-bracket. A first selection took place in the very establishment of the Scania Public Health Cohort, since the response rate was 58 %.[30] This opens up for the introduction of selection bias. However, two studies that were specifically designed to elucidate selection bias in this cohort concluded that apart from an under-representation of foreign-born individuals, the responders were not statistically different from non-responders regarding other sociodemographic characteristics,[20] nor were any signs of selection bias evident since the the risk esimates for common illnesses (including mental health) were very similar between participants and drop outs.[21, 31] In the former study, it was also noted that health care utilisation did not differ between responders and non-responders during the baseline survey year. It is therefore our assumption that selection bias did not play a major role in the findings currently obtained. Moreover, such bias could in fact give rise to an underestimation, rather than an overestimation, of the observed differences, since it seems reasonable that heavily indebted individuals for several reasons (lack of postal address, etc.) could be underrepresented in the sample, especially if their mental health was poor.

The main exposure, over-indebtedness, was based on two questions that in combination have been deemed valid by other authors[24] to identify individuals with a financial situation indicating that their income for a longer period of time was insufficient to meet their regular household bills without having other financial assets (the

For peer review only

For peer review only - http://bmjopen.bmj.com/site/about/guidelines.xhtml

definition of over-indebtedness). The prevalence of estimated over-indebtedness in our sample seems to be reasonably on par with the prevalence of over-indebtedness in the general Swedish population at the time of assessment.[32] A direct measure of over-indebtedness would be preferable, but this seems difficult to implement either by register studies or by surveys, since there is a whole range of credit forms to be covered, from regular bank loans to personal or even criminal types of credit that are very difficult to assess in a reliable and valid manner.

Our measure of precarious employment is based on relevant indicators and has been used in a recent study;[25] however, it has not been validated in relation to other existing instruments. Given that confounding from a precarious employment situation was rather weak, and considering that this factor very well could be involved in the same causal chain as the main exposure, we find it unlikely that the discussed possible misclassification had biased our finding to any degree of importance.

This study was based on self-reported data. However, the GHQ instrument has been extensively validated, and recent studies carried out in Sweden have shown that the GHQ-12 performs acceptably in detecting depression in the general population[33] and also performs acceptably in discriminating out-patients with depression and anxiety from healthy controls.[34]

Conclusions

This prospective study showed that exposure to over-indebtedness had a negative effect on vocationally active individual’s mental health. It also showed that over-indebtedness is not uncommon in the general working population, and it therefore seems to be a significant contributor to poor mental health at the population level, especially since loss of employment and secure income is likely to increase considerably in the aftermath of covid-19. Moreover, over-indebtedness was found to be clearly related to an unfavourable position with regard to societal power relations. The findings are therefore of importance not only for policy makers in the area of public

For peer review only - http://bmjopen.bmj.com/site/about/guidelines.xhtml

health and health equity, but also for policy areas that determine the risk for becoming overindebted, such as the social and judicial realms.

Funding

This work was funded by FORTE (Swedish Research Council for Health, Working Life and Welfare; Grant Number 2013-1269) and the Pufendorf Institute at Lund University (Credit Society Project). The funding institutions had no role in the design of the study, data collection, analysis and interpretation of data, or in writing of the manuscript.

Competing Interests

The authors declare that they have no conflict of interest.

Authors' Contributions

The study was concieved and designed by PÖ and CC. The analysis was done by MM. TB drafted the first version of the manuscript. All authors took part in interpreting the results and writing subsequent drafts.

Data availability statement

All data could be obtained by request to the corresponding author.

Ethics approval

All study participants gave their consent to participate in the study, which was approved by the Regional Ethical Review Board at Lund University, Sweden (Decision numbers: 1999-99; 2005-471 and 2010-392).

For peer review only

Acknowledgments

Preliminary results from this study were presented by Per-Olof Östergren at the 12th European Public Health Conference in Stockholm, 1-4 November 2017. The authors would like to thank Elizabeth Cantor-Graae for language consulting.

For peer review only

For peer review only - http://bmjopen.bmj.com/site/about/guidelines.xhtml

References

1 Alleweldt F, Kara S, Graham R, et. al. (2013). The over-indebtedness of European households: updated mapping of the situation, nature and causes, effects and initiatives for alleviating its impact – Part 1: Synthesis of findings. Retrieved from https://ec.europa.eu/info/sites/default/files/final-report-on-over-indebtedness-of-european-households-synthesis-of-findings_december2013_en.pdf. Date of access 28 January 2022.

2 OECD. (2017). Household debt (indicator) doi: 10.1787/f03b6469-en. Date of access 19 October 2017.

3 Roos J. Konsumtionsrapporten 2014. In. Edited by Archive GUPE: Centrum för konsumtionsvetenskap, Göteborg University; 2014: 54. Retrieved from: http://hdl.handle.net/2077/37778. Date of access 28 January 2022.

4 SOU 2013:72. Ut ur skuldfällan. Betänkande av 2012 års skuldsaneringsutredning. In.: Government offices of Sweden; 2013. Retrieved from: https://data.riksdagen.se/fil/A237B785-C16A-4E50-B6E1-C8CE2AD22442. Date of access 28 January 2022.

5 Simovart MA, Saare K, Sein K. Protection of Consumer Rights in SMS Loan Agreements', *European Review of Private Law* 2010;1:129-42. Retrieved from: https://kluwerlawonline.com/journalarticle/European+Review+of+Private+Law/18.1/ERPL2010006. Date of access 28 January 2022.

6 Ahlström R. Bilaga 1: Överskuldsättningens kostnader. In: *Riksrevisionen granskar.* Riksrevisionen; 2015. Retrieved from: https://www.riksrevisionen.se/download/18.78ae827d1605526e94b2db65/1518435497252/Bilaga1.pdf. Date of access 28 January 2022.

For peer review only - http://bmjopen.bmj.com/site/about/guidelines.xhtml

7 Oyesanya M, Lopez-Morinigo J, Dutta R. Systematic review of suicide in economic recession. *World J Psychiatry* 2015;5:243-54.

8 Frasquilho D, Matos MG, Salonna F, et al. Mental health outcomes in times of economic recession: a systematic literature review. *BMC Public Health* 2016;16:115.

9 Ferreira MB, de Almeida F, Soro JC, et al. On the Relation Between Over-Indebtedness and Well-Being: An Analysis of the Mechanisms Influencing Health, Sleep, Life Satisfaction, and Emotional Well-Being. *Front Psychol* 2021;12:591875. https://doi.org/10.3389/fpsyg.2021.591875

10 Warth J, Beckmann N, Puth MT, et al. Association between over-indebtedness and antidepressant use: A cross-sectional analysis. *Plos One* 2020;15:e0236393.

11 Bialowolski P, Weziak-Bialowolska D, VanderWeele TJ. The impact of savings and credit on health and health behaviours: an outcome-wide longitudinal approach. *Int J Public Health* 2019; 64:573-84.

12 Turunen E, Hiilamo H. Health effects of indebtedness: a systematic review. *BMC Public Health* 2014;14:489.

13 Selenko E, Batinic B. Beyond debt. A moderator analysis of the relationship between perceived financial strain and mental health. *Soc Sci Med* 2011;73:1725-32.

14 Sweet E, Nandi A, Adam EK et al. The high price of debt: household financial debt and its impact on mental and physical health. *Soc Sci Med* 2013;91:94-100.

15 Blomgren J, Maunula N, Hiilamo H. Over-indebtedness and chronic disease: a linked register-based study of Finnish men and women during 1995-2010. *Int J Public Health* 2016;61:535-44.

For peer review only

For peer review only - http://bmjopen.bmj.com/site/about/guidelines.xhtml

16 Hagquist CEI. Economic stress and perceived health among adolescents in Sweden. *J Adolesc Health* 1998;22:250-7.

17 Richardson T, Elliott P, Roberts R. The relationship between personal unsecured debt and mental and physical health: a systematic review and meta-analysis. *Clin Psychol Rev* 2013;33:1148-62.

18 Davies W, Montgomerie J, Wallin S. Financial melancholia: Financial health and indebtedness; 2015. Retrieved at: https://research.gold.ac.uk/id/eprint/16269/1/FinancialMelancholiaMentalHealthandIndebtedness.pdf. Date of access 28 January 2022.

19 Levinsson H, Jiborn M. Minuskontot: Ekonomiska villkor för personer med psykisk funktionsnedsättning. In.: Hjärnkoll/Nationell samverkan för Psykisk Hälsa; 2013. Retrieved from: https://lucris.lub.lu.se/ws/portalfiles/portal/6002117/4864878.pdf. Date of access 28 January 2022.

20 Carlsson F, Merlo J, Lindström M, et al. Representativity of a postal public health questionnaire survey in Sweden, with special reference to ethnic differences in participation. *Scand J Public Health* 2006;34:132-9.

21 Canivet C, Nilsson A, Björk J, et al. Assessment of selection bias due to dropouts in the follow-up of the Scania Public Health Cohort. *Scand J Public Health* 2021;49:457-64.

22 Goldberg DP, Gater R, Sartorius N, et al. The validity of two versions of the GHQ in the WHO study of mental illness in general health care. *Psychol Med* 1997;27:191-7.

23 Goldberg DP, Oldehinkel T, Ormel J. Why GHQ threshold varies from one place to another. *Psychol Med* 1998;28:915-21.

For peer review only - http://bmjopen.bmj.com/site/about/guidelines.xhtml

24 Ahnquist J, Wamala SP: Economic hardships in adulthood and mental health in Sweden. The Swedish National Public Health Survey 2009. *BMC Public Health* 2011;11:788. doi: 10.1186/1471-2458-11-788.

25 Canivet C, Bodin T, Emmelin M, et al. Precarious employment is a risk factor for poor mental health in young individuals in Sweden: a cohort study with multiple follow-ups. *BMC Public Health* 2016;16:687. doi: 10.1186/s12889-016-3358-5

26 Canivet C, Aronsson G, Bernhard-Oettel C, et al. The negative effects on mental health of being in a non-desired occupation in an increasingly precarious labour market. *SSM - Population Health* 2017; 3:516-24. https://doi.org/10.1016/j.ssmph.2017.05.009

27 Zou G. A modified poisson regression approach to prospective studies with binary data. *Am J Epidemiol* 2004;159:702-6.

28 Rothman K. Modern epidemiology. Boston: Little, Brown & Co 1986.

29 Diderichsen F, Hallqvist J, Whitehead M. Differential vulnerability and susceptibility: how to make use of recent development in our understanding of mediation and interaction to tackle health inequalities. *Int J Epidemiol* 2019;48:268-74.

30 Rosvall M, Grahn M, Modén B, et al. Hälsoförhållanden i Skåne: Folkhälsoenkät Skåne 2008: Universitetssjukhuset MAS; 2009. Retrieved from: https://utveckling.skane.se/siteassets/publikationer_dokument/folkhalsoenkat_skane_2008.pdf. Date of access 28 January 2022.

31 Nilsson A, Bonander C, Strömberg U, et al. Reweighting a Swedish health questionnaire survey using extensive population register and self-reported data for assessing and improving the validity of longitudinal associations. *PloS One* 2021;16:e0253969.

For peer review only

For peer review only - http://bmjopen.bmj.com/site/about/guidelines.xhtml

32 Hedborg A. Överskuldsättning i kreditsamhället? SOU 2013:78. Retrieved from: https://www.regeringen.se/49bb85/contentassets/7d6ed04741ab4b9badd089ed01fd3039/overskuldsattning-i-kreditsamhallet-sou-201378-hela-betankandet. Date of access 28 January 2022.

33 Lundin A, Hallgren M, Theobald H, et al. Validity of the 12-item version of the General Health Questionnaire in detecting depression in the general population. *Public Health* 2016:136:66-74.

34 Lundin A, Åhs J, Åsbring N, et al. Discriminant validity of the 12-item version of the general health questionnaire in a Swedish case-control study. *Nord J Psychiatry* 2017;71:171-9.

For peer review only

Reporting checklist for cohort study.

Based on the STROBE cohort guidelines.

Instructions to authors

Complete this checklist by entering the page numbers from your manuscript where readers will find each of the items listed below.

Your article may not currently address all the items on the checklist. Please modify your text to include the missing information. If you are certain that an item does not apply, please write "n/a" and provide a short explanation.

Upload your completed checklist as an extra file when you submit to a journal.

In your methods section, say that you used the STROBE cohortreporting guidelines, and cite them as:

von Elm E, Altman DG, Egger M, Pocock SJ, Gotzsche PC, Vandenbroucke JP. The Strengthening the Reporting of Observational Studies in Epidemiology (STROBE) Statement: guidelines for reporting observational studies.

		Reporting Item	Page Number
**Title and abstract**			
Title	#1a	Indicate the study's design with a commonly used term in the title or the abstract	3
Abstract	#1b	Provide in the abstract an informative and balanced summary of what was done and what was found	3
**Introduction**			
Background / rationale	#2	Explain the scientific background and rationale for the investigation being reported	5-7
Objectives	#3	State specific objectives, including any prespecified hypotheses	7
**Methods**			
Study design	#4	Present key elements of study design early in the paper	8-9

For peer review only

Setting	#5	Describe the setting, locations, and relevant dates, including periods of recruitment, exposure, follow-up, and data collection	8-9
Eligibility criteria	#6a	Give the eligibility criteria, and the sources and methods of selection of participants. Describe methods of follow-up.	8-9
Eligibility criteria	#6b	For matched studies, give matching criteria and number of exposed and unexposed	n/a
Variables	#7	Clearly define all outcomes, exposures, predictors, potential confounders, and effect modifiers. Give diagnostic criteria, if applicable	9-10
Data sources / measurement	#8	For each variable of interest give sources of data and details of methods of assessment (measurement). Describe comparability of assessment methods if there is more than one group. Give information separately for for exposed and unexposed groups if applicable.	9-10
Bias	#9	Describe any efforts to address potential sources of bias	10-11
Study size	#10	Explain how the study size was arrived at	8-9
Quantitative variables	#11	Explain how quantitative variables were handled in the analyses. If applicable, describe which groupings were chosen, and why	9-10
Statistical methods	#12a	Describe all statistical methods, including those used to control for confounding	

10-11

Statistical methods	#12b	Describe any methods used to examine subgroups and interactions	10-11
Statistical methods	#12c	Explain how missing data were addressed	8-9
Statistical methods	#12d	If applicable, explain how loss to follow-up was addressed	8-9
Statistical methods	#12e	Describe any sensitivity analyses	

n/a

Results

For peer review only

Participants	#13a	Report numbers of individuals at each stage of study—eg numbers potentially eligible, examined for eligibility, confirmed eligible, included in the study, completing follow-up, and analysed. Give information separately for for exposed and unexposed groups if applicable.	11 and table 1
Participants	#13b	Give reasons for non-participation at each stage	8-9
Participants	#13c	Consider use of a flow diagram	
n/a			
Descriptive data	#14a	Give characteristics of study participants (eg demographic, clinical, social) and information on exposures and potential confounders. Give information separately for exposed and unexposed groups if applicable.	11 table 1
Descriptive data	#14b	Indicate number of participants with missing data for each variable of interest	
table 1			
Descriptive data	#14c	Summarise follow-up time (eg, average and total amount)	
8			
Outcome data	#15	Report numbers of outcome events or summary measures over time. Give information separately for exposed and unexposed groups if applicable.	
11-12 table 2			
Main results	#16a	Give unadjusted estimates and, if applicable, confounder-adjusted estimates and their precision (eg, 95% confidence interval). Make clear which confounders were adjusted for and why they were included	11-13 table 2
Main results	#16b	Report category boundaries when continuous variables were categorized	9-10
Main results	#16c	If relevant, consider translating estimates of relative risk into absolute risk for a meaningful time period	
n/a			

For peer review only

Other analyses	#17	Report other analyses done—eg analyses of subgroups and interactions, and sensitivity analyses	12-13 tables 4a-b13
**Discussion**			
Key results	#18	Summarise key results with reference to study objectives	13
Limitations	#19	Discuss limitations of the study, taking into account sources of potential bias or imprecision. Discuss both direction and magnitude of any potential bias.	15-16
Interpretation	#20	Give a cautious overall interpretation considering objectives, limitations, multiplicity of analyses, results from similar studies, and other relevant evidence.	13-15
Generalisability	#21	Discuss the generalisability (external validity) of the study results	13-15, 17
**Other Information**			
Funding	#22	Give the source of funding and the role of the funders for the present study and, if applicable, for the original study on which the present article is based	17

Notes:

- 13a: 11 and table 1
- 15: 11-12 table 2
- 16a: 11-13 table 2
- 17: 12-13 tables 4a-b13 The STROBE checklist is distributed under the terms of the Creative Commons Attribution License CC-BY. This checklist was completed on 05. February 2022 using https://www.goodreports.org/, a tool made by the EQUATOR Network in collaboration with Penelope.ai

For peer review only

BMJ Open

Selling one’s future: Over-indebtedness and the risk of poor mental health and the role of precarious employment – results from the Scania Public Health Cohort, Sweden

Journal:	*BMJ Open*
Manuscript ID	bmjopen-2022-061797.R1
Article Type:	Original research
Date Submitted by the Author:	07-Sep-2022
Complete List of Authors:	Östergren, Per-Olof; Lund University, Department of Clinical Sciences Malmö<br>Bodin, Theo; Karolinska Institutet; Center for Occupational and Environmental Medicine<br>Canivet, Catarina ; Lund University, Dept of Clinical Sciences Malmo<br>Moghaddassi, Mahnaz; Lund University, Department of Clinical Sciences Malmö<br>Vilhelmsson, Andreas; Lund University, Department of Laboratory Medicine
**Primary Subject Heading**:	Epidemiology
Secondary Subject Heading:	Mental health, Public health
Keywords:	MENTAL HEALTH, EPIDEMIOLOGY, Health policy < HEALTH SERVICES ADMINISTRATION & MANAGEMENT, PUBLIC HEALTH, Public health < INFECTIOUS DISEASES

SCHOLARONE™ Manuscripts

I, the Submitting Author has the right to grant and does grant on behalf of all authors of the Work (as defined in the below author licence), an exclusive licence and/or a non-exclusive licence for contributions from authors who are: i) UK Crown employees; ii) where BMJ has agreed a CC-BY licence shall apply, and/or iii) in accordance with the terms applicable for US Federal Government officers or employees acting as part of their official duties; on a worldwide, perpetual, irrevocable, royalty-free basis to BMJ Publishing Group Ltd ("BMJ") its licensees and where the relevant Journal is co-owned by BMJ to the co-owners of the Journal, to publish the Work in this journal and any other BMJ products and to exploit all rights, as set out in our licence.

The Submitting Author accepts and understands that any supply made under these terms is made by BMJ to the Submitting Author unless you are acting as an employee on behalf of your employer or a postgraduate student of an affiliated institution which is paying any applicable article publishing charge ("APC") for Open Access articles. Where the Submitting Author wishes to make the Work available on an Open Access basis (and intends to pay the relevant APC), the terms of reuse of such Open Access shall be governed by a Creative Commons licence – details of these licences and which Creative Commons licence will apply to this Work are set out in our licence referred to above.

Other than as permitted in any relevant BMJ Author's Self Archiving Policies, I confirm this Work has not been accepted for publication elsewhere, is not being considered for publication elsewhere and does not duplicate material already published. I confirm all authors consent to publication of this Work and authorise the granting of this licence.

For peer review only

Selling one's future: Over-indebtedness and the risk of poor mental health and the role of precarious employment – results from the Scania Public Health Cohort, Sweden

Per-Olof Östergren[1], Theo Bodin[2,3], Catarina Canivet[1], Mahnaz Moghaddassi[1], Andreas Vilhelmsson[4]

1. Social Medicine and Global Health, Department of Clinical Sciences Malmö, Lund University, Lund, Sweden
2. Unit of Occupational Medicine, Institute of Environmental Medicine, Karolinska Institutet, Stockholm, Sweden
3. Center for Occupational and Environmental Medicine, Stockholm Region, Stockholm, Sweden
4. Division of Occupational and Environmental Medicine, Department of Laboratory Medicine, Lund University, Lund, Sweden

Corresponding author: Per-Olof Östergren, per-olof.ostergren@med.lu.se, +46 705160092

Word count abstract: 300

Word count article: 4224

For peer review only

For peer review only - http://bmjopen.bmj.com/site/about/guidelines.xhtml

Abstract

OBJECTIVES: The credit market has expanded rapidly, increasing the risk of over-indebtedness among those who lack secure employment or adequate income, an issue of concern in the Covid-19 aftermath. We investigated the role of over-indebtedness for developing poor mental health, and whether this impact is modified by age, gender, educational level, or being in precarious employment.

METHODS: This is a cohort study using data from the Swedish Scania Public Health Cohort, based on individuals randomly selected from the general adult population in Scania, southern Sweden, initiated in 1999/2000 (response rate 58%) with follow-ups in 2005 and 2010. Over-indebtedness was assessed by combining information on cash margin and difficulty in paying household bills. Mental health was assessed by General Health Questionnaire-12. Those with poor mental health at baseline were excluded, and the analyses were further restricted to vocationally active individuals with complete data on main variables, resulting in 1256 men and 1539 women.

RESULTS: Over-indebtedness was more common among women, among persons with a low educational level, born abroad, and with a precarious employment at baseline. The age-adjusted incidence rate ratio (IRR) for poor mental health in 2010 among individuals exposed to over-indebtedness in 1999/2000 or 2005 was 2.2 (95% CI: 1.7–2.8). Adjusting for educational level, country of origin, and precarious employment in 1999/2000 or 2005, yielded an IRR of 2.0; 95% CI: 1.6–2.6. An interaction analysis indicated that a high level of education may act synergistically with over-indebtedness, regarding poor mental health among men.

CONCLUSIONS: Over-indebtedness was related to unfavourable societal power relations, regarding social class, gender, and foreign birth. Precarious employment was independently linked to poor mental health and may also mediate the effect by over-indebtedness. The Covid-19 pandemic might entail increased over-indebtedness, which should be acknowledged in policies aiming at buffering social effects of the pandemic.

For peer review only - http://bmjopen.bmj.com/site/about/guidelines.xhtml

For peer review only

Strengths and limitations of this study

- This was a prospective cohort study with 2795 vocationally active men and women randomly selected from the general adult population.
- The exposure and outcome variables have been used and validated in previous studies.
- Detailed information concerning employment history and details regarding current empolyment for each individual was used for determining precarious empolyment status.
- Non-response and missing data among respondents may have caused some selection bias.
- We lacked access to an objective measure regarding over-indebtedness, which may have caused misclassification.

For peer review only

Introduction

Credit plays a paramount role in modern economies, since it increases the possibility to make investments that can generate future financial and other gains and to allocate lifetime income in a more optimal relation to need. From a health perspective, such resources are very likely related to opportunities for improved health over the life-course. However, when the credit market targets those who cannot make ends meet, this can cause increasing indebtedness, which can lead to poorer health, both due to loss of material and other resources and to exposure to growing stress and worries about one´s loss of control over the household economy. Thus, the proportion of individuals who are prone to over-indebtedness will most likely expand considerably in the aftermath of the Covid-19 pandemic. Moreover, indebtedness could be expected to have a differential effect on health, much depending on factors such as socioeconomic position, country of birth (i.e. migration background), and age.

The credit market has expanded over the last decades leading to a build-up of consumer debt. However, it was not until the financial crisis in 2007-2008 that an increased proportion of households that had difficulties making ends meet was noted in the EU.[1] As of 2016, the household debt in relation to disposable income was more than twice as high in the OECD compared to 1996.[2] Measured as share of GDP, the indebtedness of Swedish households has doubled since the 1990s.[3] According to public reports, the number of over-indebted individuals in Sweden amounts to between 6 and 18 per cent of the population, depending on definition. The subsequent yearly societal cost is estimated to 30 billion SEK.[4] The credit market has also changed in terms of type of credit providers and types of 'products', e.g. so called 'pay-day loans' as a temporary financial solution when financial means are insufficient to cover daily cost of living because of inadequate income and lack of savings.[5] These types of loans have at the same time become very accessible via the Internet and mobile phones. Since the interest rates and fees generally are very high for these loans, this could

For peer review only

For peer review only - http://bmjopen.bmj.com/site/about/guidelines.xhtml

quickly lead to a spiral of indebtedness where the loan-taker in practice ends up owing the lender a substantial proportion of his/her future income.[6]

Over-indebtedness can be defined as a situation, persisting for a sustained period of time, in which daily cost of living exceeds income, and in which the imbalance cannot be compensated by savings. This will inevitably lead to use of credit in one way or another, the cost of which will add to the discrepancy between expenditure on the one side and income and savings on the other. This situation could represent an emerging significant stressor and determinant of ill health, and thereby constitute a source of health inequality that is likely to be overlooked in research on income or wealth and health, since information concerning over-indebtedness can not easily be retrieved from registers on income, wealth, or social transfers.

A growing number of scientific studies on the indirect and direct association between over-indebtedness and health have been published in the scientific literature. Systematic reviews have provided support for the assumption that the financial recession has been associated with an increase in suicide rates in high-income countries such as Europe and North America.[7] Further, socioeconomic mediators such as unemployment, income decline, and unmanageable debts are significantly associated with poor mental wellbeing, increased rates of common mental disorders, substance-related disorders, and suicidal behaviours.[8-11] Individuals who cannot make their loan payments have been found to suffer from depression and have suicidal ideation more often compared to those without such problems.[12] Another study investigating predictors of mental health in people on the verge of bankruptcy found that perceived financial strain was an important predictor of poor mental health.[13] However, most of the studies have been cross-sectional, and more longitudinal studies are needed to corroborate the mentioned findings.

High debt relative to available assets has also been associated with poorer self-assessed general health, higher diastolic blood pressure, obesity, poorer health-related behaviour, and myocardial

For peer review only

infarction.[14] The effect of over-indebtedness on health has been found to be stronger for women than for men.[15] In a life-course perspective, poor self-rated health has been found to be even more prevalent among those who have experienced economic stress both early in life and later on.[16]

According to a systematic review and meta-analysis examining the relationship between personal debt and mental health,[17] ten epidemiological studies with nationally representative samples of the general population were found, whereof seven came from the UK and nine out of ten were cross-sectional. Unsurprisingly, very high odds ratios for different mental health outcomes were found. In many cases the causality pattern could be described as a vicious circle, which could start either by a decrease in income or in health. Poor mental health provoked by over-indebtedness tends to increase the risk of further financial problems and so forth.[18] Moreover, over-indebtedness or coping with excessive credit is associated with subjective distress such as shame, anxiety, and apathy.[19] The mentioned process could involve mediating mechanisms, as well as situations where certain exposures modify the effect of each other, such that already vulnerable groups, e.g. individuals with low education or in a precarious employment situation, are hit more severely by over-indebtedness in terms of resulting poor mental health.

To the best of our knowledge, evidence regarding the role of precarious employment as a mediator or modifier of the impact of over-indebtedness on mental health is lacking.

In summary, previous research relies heavily on cross-sectional data from the UK, why studies from other national contexts are needed. Moreover, because of the plausible bidirectional causal pathways, prospective studies of long duration with several assessments of both types of variables, over-indebtedness as well as mental health, are preferable. Also, as mentioned above, little is known about the role of precarious employment and the differential health effects across sociodemographic groups.

For peer review only - http://bmjopen.bmj.com/site/about/guidelines.xhtml

Objectives

The aim of the study was to investigate the role of over-indebtedness for the development of poor mental health in a longitudinal perspective, and whether this impact is modified by age, gender, educational level, housing tenure, or being in a precarious employment situation.

Methods

Study population

The present cohort was established in 1999/2000 and followed up in 2005 and 2010.[20] At baseline, a postal questionnaire was sent out to 25 000 men and women, 18–80 years old. These individuals were randomly selected from the population register, such that equal representation was achieved from all municipalities in the county of Scania (population 1.3 million), Sweden. In total, 13589 out of 23437 eligible individuals returned a completed questionnaire (response rate 58%). All of those who responded at baseline and still residing in the county were invited to follow-up after five and ten years. Out of 12002 respondents alive and still living in the region after 10 years, there were 8206, i.e. 68%, who also participated in the 2005 and 2010 inquiries.

A comparison between these 8206 respondents and the corresponding Scania general population at baseline [20] showed that younger individuals, men, and individuals with a low educational level were slightly underrepresented among respondents. However, a recently published study from this cohort, showed very little proof of selection bias regarding common health outcomes, includung mental health.[21]

For peer review only

For peer review only - http://bmjopen.bmj.com/site/about/guidelines.xhtml

Since one aim of the study was to look into the relationship between precarious employment and over-indebtedness and mental health, we firstly excluded individuals who were not vocationally active, i.e. at baseline were retired, on disability pension, or on long-term sick leave, and also those who answered that they did not wish to work a year from now (N=1744). Out of the remaining 6462 persons, those with lacking data on employment precariousness (N=2479) were excluded; out of these, 1106 were 55–80 years old at baseline, i.e. were qualified for old-age retirement during the studied window of time. Further, we excluded individuals who lacked baseline data regarding the variables included in the multivariable analyses (rather than assigning them as 'internal' missing); i.e. housing tenure (N=159) or educational level (N=158), also those lacking baseline and 2005 information on over-indebtedness (N=249), furthermore those who lacked information regarding mental health at all follow-ups (N=161). Lastly, in order to reduce the influence of reverse causation, we also excluded individuals with poor mental health at baseline (N=1563), instead of controlling for this in the multivariable analysis. Thus, the final study population consisted of 2795 individuals, 1256 men and 1539 women.

Outcome variable – poor mental health

Mental health was measured at all three time points of assessment, using the 12-item version of the General Health Questionnaire (GHQ-12). We used the 0-0-1-1 scoring method (range 0–12) recommended by the creators of the instrument, [22] with poor mental health or 'GHQ-caseness' determined by the population mean and defined as a score of 2 or higher.[23]

Over-indebtedness

Two items have been used previously in several Swedish studies to evaluate economic hardship, namely inability to meet expenses and lacking cash reserves.[24] For the former we used the question 'How often during the last 12 months have you had problems paying your bills?' and for the latter, 'In

For peer review only - http://bmjopen.bmj.com/site/about/guidelines.xhtml

case of an unforeseen emergency, do you have the capacity to raise 12000 SEK *(approximately 1100 EUR)* in a week's time?' The same questions were asked in 1999/2000, 2005, and 2010, but the specified amount was raised to 14000 SEK, approximately 1300 EUR, in the 2010 survey. Over-indebtedness was defined as affirming 'about half of the months' (or more often) on the first question and answering 'no' on the second.

Other variables

Age was classified into four groups, except in the interaction analysis, where it was dichotomised, and in the multivariate analysis, where it was used as a continuous variable. Country of origin was recorded as 'born in Sweden' (yes/no). Educational level at baseline was determined by the self-reported total years of formal education and classified into four groups. Housing tenure was dichotomised as either owned or rented residence. For employment precariousness, we used a dichotomous variable, based on a combination of data on present unemployment, episode of involuntary unemployment during the past three years (no/yes), currently temporarily vs. permanently employed, and perceived job insecurity.[25, 26]

Statistical methods

The relationships between background factors and poor mental health in 2010 are presented as percentages and age-adjusted incidence rate ratios (IRR), which are a good estimate of relative risks, using a modified Poisson regression model with robust standard errors.[27] In the multivariate analysis, over-indebtedness was tested against the outcome with the stepwise addition of educational level, country of origin, and precarious employment.

The tests for effect modification were performed with simple dummy variables. The synergy indices were calculated as proposed by Rothman.[28] according to which a synergy index > 1 may indicate a

For peer review only

synergistic effect, and a synergy index < 1 an antagonistic effect between two exposure variables. In the interaction analysis, educational level was dichotomised as ’12 years or less’ versus ’13 years or more’. There was a negative relationship between educational level and over-indebtedness (see Results), while the relationship with the outcome variable poor mental health at follow-up was positive (see Results), and therefore a high educational level was designated as exposure.

Two standard statistical analysis programmes were used, IBM SPSS Statistics for Windows, Version 22.0, Armonk, NY: IBM Corp. and Stata Statistical Software: Release 12, College Station, TX: StataCorp LP.

Patient and public involvement statement

Patient or public involvement in the design, reporting or dissemination of results was not possible.

Results

Table 1 shows the prevalence of over-indebtedness in the total study population at baseline and also separately by gender. Overall, the prevalence of overindebted individuals was 4.2%, but with a clear difference between men and women (2.8 versus 5.3%, respectively). The only clear age-related difference in over-indebtedness consisted of a low prevalence in participants aged 55 and above. Over-indebtedness was more common in those with an educational level of 10–12 years, vs. those with less or with more years. When educational level was dichotomised as ’12 years or less’ vs. ’13 years or more’, 5.5% of those with a low educational level were overindebted at baseline, vs. 2.7% of those with a high educational level (data not shown in tables). Over-indebtedness was three times as common among those born in another country than Sweden. As perhaps expected, over-indebtedness was almost four times as prevalent among those who reported a precarious employment situation and

For peer review only - http://bmjopen.bmj.com/site/about/guidelines.xhtml

among those who rented their dwelling (compared to those who owned it). The gender pattern was largely similar for all factors.

For peer review only

Table 1

Background characteristics at baseline in 1999/2000 of 1256 men and 1539 women from the Scania Public Health Cohort, in relation to over-indebtedness.

		All				Men				Women			
		Total	No	Yes[a]	% Yes	Total	No	Yes	% Yes	Total	No	Yes	% Yes
Total		2795	2679	116	4.2	1256	1221	35	2.8	1539	1458	81	5.3
Age	18–24	207	196	11	5.3	96	92	4	4.2	111	104	7	6.3
	25–34	621	596	25	4.0	278	272	6	2.2	343	324	19	5.5
	35–44	750	710	40	5.3	324	314	10	3.1	426	396	30	7.0
	45–54	966	930	36	3.7	430	416	14	3.3	536	514	22	4.1
	55–63	251	247	4	1.6	128	127	1	0.8	123	120	3	2.4
Educational level	9 y or less	502	484	18	3.6	265	256	9	3.4	237	228	9	3.8
	10–12	954	892	62	6.5	437	416	21	4.8	517	476	41	7.9
	13–14	633	609	24	3.8	253	250	3	1.2	380	359	21	5.5
	15 y or more	706	694	12	1.7	301	299	2	0.7	405	395	10	2.5
Born in Sweden	Yes	2588	2494	94	3.6	1163	1136	27	2.3	1425	1358	67	4.7
	No	207	185	22	10.6	93	85	8	8.6	114	100	14	12.3
Precarious employment	No	2051	2004	47	2.3	943	926	17	1.8	1108	1078	30	2.7
	Yes	744	675	69	9.3	313	295	18	5.8	431	380	51	11.8
Housing tenure	Owned	2176	2112	64	2.9	990	969	21	2.1	1186	1143	43	3.6
	Rented	619	567	52	8.4	266	252	14	5.3	353	315	38	10.8

[a] Over-indebted at baseline

For peer review only

Table 2 shows the results of the longitudinal analysis, where poor mental health at follow-up in 2010 was used as the outcome. The age-adjusted risk for poor mental health at follow-up in overindebted individuals, measured as incidence rate ratio (IRR), was 2.2 (95% CI: 1.7–2.8) in the entire group, 2.6 (1.8–3.9) for men, and 1.9 (1.4–2.6) for women. The risk for poor mental health was four times higher in those who were 18–24 years at baseline, with an IRR of 4.1 (2.3–7.4), and decreased successively with increasing age, compared to those who were 55 or older at baseline, which was used as the reference group. That pattern was similar in men and women. Those with an educational level of 13–14 years at baseline had a slightly higher risk of poor mental health at follow-up (IRR=1.4; 95% CI: 1.01–2.0) than those with 9 years or less, which was used as the reference. When educational level was dichotomised, 15.1% of those with a high educational level had poor mental health at follow-up vs. 13.9% of those with a low educational level (data not shown in tables). Foreign birth was related to poor mental health in men only, with an IRR of 1.6 (1.01–2.6). Having a precarious employment situation during the period 1999/2000 to 2005 was statistically significantly related to poor mental health in 2010 (IRR= 1.4; 95% CI:1.2–1.7), albeit also here with a gender difference; the IRR for men was 1.8 (1.3–2.4) and for women 1.2 (0.95–1.5). Housing tenure (rented dwelling) at baseline was not related to poor mental health in 2010.

For peer review only

Table 2

Background characteristics from 1999/2000 through 2005 in relation to poor mental health in 2010.

		All				Men				Women			
		N cases poor mental health [a]	% cases poor mental health	IRR [b]	95% CI [c]	N cases poor mental health	% cases poor mental health	IRR	95% CI	N cases poor mental health	% cases poor mental health	IRR	95% CI
Total		404	14.5			144	11.5			260	16.9		
Over-indebtedness in 1999/2000 or 2005, at least once	No	348	13.3	1		124	10.4	1		224	15.7		1
	Yes	56	31.8	2.2	1.7–2.8	20	31.3	2.6	1.8–3.9	36	32.1	1.9	1.4–2.6
Age in 1999/2000	18–24	44	21.3	4.1	2.3–7.4	17	17.7	5.7	2.0–16.3	27	24.3	3.3	1.6–6.8
	25–34	125	20.1	3.9	2.2–6.8	46	16.5	5.3	2.0–14.4	79	23.0	3.1	1.6–6.1
	35–44	114	15.2	2.9	1.7–5.1	41	12.7	4.0	1.5–11.1	73	17.1	2.3	1.2–4.5
	45–54	108	11.2	2.2	1.2–3.8	36	8.4	2.7	0.97–7.4	72	13.4	1.8	0.9–3.6
	55–63	13	5.2	1		4	3.1	1		9	7.3	1	
Educational level in 1999/2000	9 y or less	47	9.4	1		18	6.8	1		29	12.2	1	
	10–12	155	16.2	1.3	0.9–1.8	61	14.0	1.4	0.8–2.5	94	18.2	1.1	0.8–1.7
	13–14	107	16.9	1.4	1.01–2.0	27	10.7	1.2	0.7–2.1	80	21.1	1.4	0.9–2.1
	15 y or more	95	13.5	1.2	0.8–1.6	38	12.6	1.4	0.8–2.5	57	14.1	0.9	0.6–1.4
Born in Sweden	Yes	369	14.3	1		128	11.0	1		241	16.9	1	
	No	35	16.9	1.2	0.9–1.6	16	17.2	1.6	1.01–2.6	19	16.7	1.0	0.6–1.5
Precarious employment in 1999/2000 or 2005, at least once	No	211	12.0	1		69	8.5	1		142	15.0	1	
	Yes	193	18.6	1.4	1.2–1.7	75	16.7	1.8	1.3–2.4	118	20.0	1.2	0.95–1.5
Housing tenure in 1999/2000	Owned	292	13.4	1		101	10.2	1		191	16.1	1	
	Rented	112	18.1	1.1	0.9–1.4	43	16.2	1.3	0.9–1.9	69	19.5	1.0	0.8–1.3

[a] Poor mental health is defined as GHQ-caseness; [b] IRR, incidence rate ratio, age-adjusted; [c] CI, confidence interval

For peer review only

For peer review only - http://bmjopen.bmj.com/site/about/guidelines.xhtml

Table 3 shows the effect on the risk estimate for poor mental health at follow-up when exposed to over-indebtedness during the period 1999/2000 to 2005, with a step-wise introduction of the main covariates. In the final step, when precarious employment situation during 1999/2000 to 2005 was introduced into the analysis, the risk estimate for poor mental health regarding exposure to over-indebtedness 1999/2000 to 2005 decreased about 10% for the entire group. The decrease in IRR in the fully adjusted model was slightly larger for men than for women. As a general remark, we find it possible that this is a case of over-adjustment, since all the introduced covariates could be included in the same causal chains, as well as in separate ones, and we have no way to disentangle this.

For peer review only

Table 3

Over-indebtedness at least once in 1999/2000 or 2005 in relation to the outcome poor mental health at follow-up in 2010, with forward stepwise addition of potential confounders; educational level in 1999/2000, country of origin, and precarious employment at least once in 1999/2000 or 2005.

			Model 1 Age-adjusted		Model 2 Model 1 + adjusted for educational level in 1999/2000		Model 3 Model 2 + adjusted for country of origin		Model 4 Model 3 + adjusted for precarious employment in 1999/2000 or 2005	
			IRR[a]	95% CI[b]	IRR	95% CI	IRR	95% CI	IRR	95% CI
All	Over-indebtedness	Yes versus no	2.2	1.7–2.8	2.2	1.7–2.8	2.2	1.7–2.8	2.0	1.6–2.6
	Educational level	9 years or less			1		1		1	
		10–12 years			1.2	0.9–1.7	1.2	0.9–1.7	1.3	0.9–1.8
		13–14 years			1.4	1.03–2.0	1.4	1.03–2.0	1.5	1.1–2.1
		15 years or more			1.2	0.9–1.7	1.2	0.9–1.7	1.3	0.9–1.8
	Born in Sweden	No versus yes					1.1	0.8–1.4	1.0	0.7–1.4
	Precarious employment	Yes versus no							1.3	1.1–1.6
Men	Over-indebtedness	Yes versus no	2.6	1.8–3.9	2.7	1.8–4.2	2.6	1.7–4.0	2.2	1.4–3.5
	Educational level	9 years or less			1		1		1	
		10–12 years			1.4	0.8–2.4	1.4	0.8–2.4	1.4	0.8–2.4
		13–14 years			1.2	0.7–2.2	1.3	0.7–2.2	1.2	0.7–2.2
		15 years or more			1.6	0.9–2.8	1.6	0.9–2.7	1.6	0.9–2.8
	Born in Sweden	No versus yes					1.3	0.8–2.1	1.2	0.7–1.9
	Precarious employment	Yes versus no							1.6	1.2–2.3
Women	Over-indebtedness	Yes versus no	1.9	1.4–2.6	1.9	1.4–2.5	1.9	1.4–2.6	1.8	1.4–2.5
	Educational level	9 years or less			1		1		1	
		10–12 years			1.1	0.7–1.7	1.1	0.7–1.7	1.1	0.8–1.7
		13–14 years			1.4	0.9–2.1	1.4	0.9–2.1	1.4	0.9–2.1
		15 years or more			1.0	0.6–1.5	1.0	0.6–1.5	1.0	0.6–1.5
	Born in Sweden	No versus yes					0.9	0.6–1.4	0.9	0.6–1.3
	Precarious employment	Yes versus no							1.1	0.9–1.4

[a] IRR, incidence rate ratio; [b] CI, confidence interval

For peer review only

As it is of considerable interest to investigate whether there are certain segments of the population that are more vulnerable concerning the risk of developing poor mental health when exposed to over-indebtedness, we examined this concerning age and level of education. As seen in Table 4A, there was no indication of a synergistic effect of young age and over-indebtedness on the risk of poor mental health at follow-up. However, as seen in Table 4B, while there was likewise no interaction between a high educational level and over-indebtedness in women, a higher risk for poor mental health was seen in men with this particular combination of risk factors. The IRR for men was 3.8 (95% CI: 1.8–8.0), and the resulting synergy index 1.8.

For peer review only

For peer review only - http://bmjopen.bmj.com/site/about/guidelines.xhtml

Table 4A

Interaction analyses, by gender, with synergy indices regarding age group in 1999/2000 and over-indebtedness at least once in 1999/2000 or 2005, in relation to poor mental health in 2010.

	All					Men					Women				
Age group in 1999/2000 (18–34 vs 35–63) and over-indebtedness in 1999/2000 or 2005	N	% cases poor mental health	IRR[a]	95% CI[b]	SI[c]	N	% cases poor mental health	IRR[a]	95% CI[b]	SI[c]	N	% cases poor mental health	IRR[a]	95% CI[b]	SI[c]
Older & No over-indebtedness	1859	11.0	1			844	8.4	1			1015	13.2	1		
Older & Over-indebtedness	108	27.8	2.5	1.8–3.5		38	26.3	3.1	1.8–5.6		70	28.6	2.2	1.5–3.2	
Younger & No over-indebtedness	760	18.8	1.7	1.4–2.1		348	15.2	1.8	1.3–2.5		412	21.8	1.7	1.3–2.1	
Younger & Over-indebtedness	68	38.2	3.5	2.5–4.8	1.1	26	38.5	4.6	2.7–7.8	1.2	42	38.1	2.9	1.9–4.4	1.0
	2795					1256					1539				

[a] IRR, incidence rate ratio; [b] CI, confidence interval; [c] SI, synergy index

For peer review only

For peer review only - http://bmjopen.bmj.com/site/about/guidelines.xhtml

Table 4B
Interaction analyses, by gender, with synergy indices regarding educational level in 1999/2000 and over-indebtedness at least once in 1999/2000 or 2005, in relation to poor mental health in 2010.

	All					Men					Women				
Educational level in 1999/2000 and over-indebtedness in 1999/2000 or 2005	N	% cases poor mental health	IRR[a]	95% CI[b]	SI[c]	N	% cases poor mental health	IRR[a]	95% CI[b]	SI[c]	N	% cases poor mental health	IRR[a]	95% CI[b]	SI[c]
Low & No over-indebtedness	1336	12.4	1			649	9.9	1			687	14.8	1		
Low & Over-indebtedness	120	30.0	2.2	1.6–3.0		53	28.3	2.5	1.5–4.1		67	31.3	2.0	1.3–2.9	
High & No over-indebtedness	1283	14.2	1.1	0.9–1.3		543	11.0	1.1	0.8–1.5		740	16.5	1.1	0.9–1.4	
High & Over-indebtedness	56	35.7	2.6	1.8–3.8	1.2	11	45.5	3.8	1.8–8.0	1.8	45	33.3	2.1	1.3–3.2	1.0
	2795					1256					1539				

[a] IRR, age-adjusted incidence rate ratio; [b] CI, confidence interval; [c] SI, synergy index

For peer review only

Discussion

This study confirmed that vocationally active individuals who are exposed to over-indebtedness have an increased risk of developing poor mental health. Age-adjusted estimates showed that the risk was almost tripled for men and doubled for women in this general adult population sample of vocationally active individuals (18–65 years). Considering that about 4 % of all individuals in the cohort were exposed to over-indebtedness at baseline and probably substantially more during the follow-up period, over-indebtedness should be regarded as an important general population risk factor for poor mental health. This is pertinent especially in the aftermath of the Covid-19 pandemic, during which the number of vulnerable individuals in the adult population is likely to increase considerably.

At the same time, especially from a health equity perspective, it must be acknowledged that the prevalence and to some extent the impact of exposure to over-indebtedness in the population depend on sociodemographic circumstances. Previous reseaerch provides few examples of how this could be integrated into a coherent theoretical framework regarding health equity. We speculate that the reason that younger individuals are more exposed to over-indebtedness than middle-aged or older ones may partly have a 'life-course' explanation (mortgage loans are taken early in life and paid off over time), but there is also a generational effect due to the expansion of credit markets over time. For the other group disparities in over-indebtedness, reasons must be sought elsewhere. Women were more exposed than men, foreign-born more than those born in Sweden, and those with a low educational level more than those with a high level. In short, it seems that the risk for over-indebtedness follows all the main societal power structures, i.e. gender, social class, and ethnicity. Therefore, it is an important factor to consider when formulating and implementing policies for promoting health equity.

Regarding socioeconomic factors that logically should be strongly related to over-indebtedness, i.e. precarious employment and housing ownership (i.e. non-ownership), we found that both these factors were strongly related to over-indebtedness, but that only precarious employment was also related to the risk of developing poor mental

For peer review only - http://bmjopen.bmj.com/site/about/guidelines.xhtml

health during our observation period. However, when we performed an analysis to control for confounding from precarious employment, the impact of over-indebtedness on poor mental health was only moderately reduced. Considering a bidirectional relation between over-indebtedness and poor mental health, the choice to exclude individuals with poor mental health at baseline could have yielded an underestimation of the true association between the variables.

Health inequality could be the result of both differential exposure and differential impact when once exposed.[29] From the discussion above, it seems obvious that there is a clear case of differential exposure regarding over-indebtedness and sociodemographic factors. When we tested by means of two-way interactions whether we in addition could find any evidence for a differential impact, we concluded that this was not the case regarding age (Table 4A). We noted, however, that the combination of a high educational level and over-indebtedness (Table 4B) was associated with a synergy index of 1.0 in women, whereas the corresponding index in men was 1.8, thus indicating that these factors may act synergistically in a detrimental way for men. One could speculate that over-indebtedness may be especially hard to endure for a well-educated man, since over-indebtedness implies that one is financially not 'in control', whereas gender-based stereotypes of masculinity imply the opposite. Added to that might be the frustration caused by having invested many years in education and not being 'rewarded' by economic stability. Nevertheless, this finding relies on an analysis in which the number of exposed men is small, why the confidence intervals are large. Therefore, this result needs to be replicated in other research.

As shown in many studies as well as in this one, the basic power relations in society, among which would be those determined by gender, social class, and ethnicity, show a strong relation to the risk of becoming over-indebted. This is not surprising, because all the above mentioned power relations determine access to factors that could avoid a short- or long-term situation where credit is needed for securing stable living conditions. Not being able to do so will induce both a loss of control and a loss of material resources, situations that have been shown to increase individuals' susceptibility to poor health development, especially poor mental health. However, the

For peer review only

'Faustian deal' of using credit in this situation could make things worse, since a spiral of failing credit and declining mental health could place an individual in a situation where he or she in fact has sold a considerable part of future earnings to a moneylender institute. The possibility of having such debts cancelled in a regulated way, which is open to business enterprises through the legal bankruptcy framework, is very restricted for individuals in the Swedish context. This is particularly problematic in a health equity perspective because of the strong association between societal power structures and the risk of becoming overindebted, all of which could be magnified in the wake of the Covid-19 pandemic.

The results of this study are therefore very relevant for policy, not only regarding public health and health equity, but also for policy formulation and implementation in the judicial and social realms. A specific policy recommendation is to expand the possibilities to write off debt for the individual, in a similar way that now is possible for business enterprises, in order to prevent a vicious circle of poor mental health and increasing debt, which seems maleficent for all involved stakeholders. Because of the growing problem with over-indebtedness, it seems urgent to develop the information systems concerning individual's and household's financial situation, in a manner that high quality information becomes available, not only concerning income but equally concerning financial assets and debt. This, especially since increasing inequality appears to be better mirrored by the latter, rather than the former.

For peer review only

Strengths and weaknesses

This study utilises a prospective design using a large randomly selected general population sample of individuals in the occupationally active age-bracket in southern Sweden. A first selection took place in the very establishment of the Scania Public Health Cohort, since the response rate was 58 %.[30] This opens up for the introduction of selection bias. However, two studies that were specifically designed to elucidate selection bias in this cohort concluded that apart from an under-representation of foreign-born individuals, the responders were not statistically different from non-responders regarding other sociodemographic characteristics,[20] nor were

For peer review only - http://bmjopen.bmj.com/site/about/guidelines.xhtml

any signs of selection bias evident since the risk esimates for common illnesses (including mental health) were very similar between participants and drop outs.[21, 31] In the former study, it was also noted that health care utilisation did not differ between responders and non-responders during the baseline survey year. Moreover, such bias could in fact give rise to an underestimation, rather than an overestimation, of the observed differences, since it seems reasonable that heavily indebted individuals for several reasons (lack of postal address, etc.) could be underrepresented in the sample, especially if their mental health was poor.

The main exposure, over-indebtedness, was based on two questions that in combination have been deemed valid by other authors[24] to identify individuals with a financial situation indicating that their income for a longer period of time was insufficient to meet their regular household bills without having other financial assets (the definition of over-indebtedness). We believe that this measure might be more objective, since it builds on two factual circumstances, than measures solely based on a subjective assessment of financial stress. The prevalence of estimated over-indebtedness in our sample seems to be reasonably on par with the prevalence of over-indebtedness in the general Swedish population at the time of assessment.[32] A direct measure of over-indebtedness would be preferable, but this seems difficult to implement either by register studies or by surveys, since there is a whole range of credit forms to be covered, from regular bank loans to personal or even criminal types of credit that are very difficult to assess in a reliable and valid manner.

Our measure of precarious employment is based on relevant indicators and has been used in a recent study;[25] however, it has not been validated in relation to other existing instruments. Given that confounding from a precarious employment situation was rather weak, and considering that this factor very well could be involved in the same causal chain as the main exposure, we find it unlikely that the discussed possible misclassification had biased our finding to any degree of importance. Although we have assessed potential confounding from main socioeconomic background factors known to be linked to over-indebtedness, such as gender, educational level, age, and migration status, we cannot fully rule out residual confounding from other factors.

For peer review only - http://bmjopen.bmj.com/site/about/guidelines.xhtml

This study was based on self-reported data. However, the GHQ instrument has been extensively validated, and recent studies carried out in Sweden have shown that the GHQ-12 performs acceptably in detecting depression in the general population[33] and also performs acceptably in discriminating out-patients with depression and anxiety from healthy controls.[34]

Conclusions

This prospective study showed that exposure to over-indebtedness had a negative effect on vocationally active individual's mental health. It also showed that over-indebtedness is not uncommon in the general working population, and it therefore seems to be a significant contributor to poor mental health at the population level, especially since loss of employment and secure income is likely to increase considerably in the aftermath of Covid-19. Moreover, over-indebtedness was found to be clearly related to an unfavourable position with regard to societal power relations. The findings are therefore of importance not only for policy makers in the area of public health and health equity, but also for policy areas that determine the risk for becoming over-indebted, such as the social and judicial realms.

Funding

This work was funded by FORTE (Swedish Research Council for Health, Working Life and Welfare; Grant Number 2013-1269) and the Pufendorf Institute at Lund University (Credit Society Project). The funding institutions had no role in the design of the study, data collection, analysis and interpretation of data, or in writing of the manuscript.

Competing Interests

The authors declare that they have no conflict of interest.

For peer review only - http://bmjopen.bmj.com/site/about/guidelines.xhtml

Authors' Contributions

The study was concieved and designed by PÖ, CC and AV. The analysis was done by MM. TB drafted the first version of the manuscript. All authors took part in interpreting the results and writing subsequent drafts.

Data availability statement

All data could be obtained by request to the corresponding author.

Ethics approval

All study participants gave their consent to participate in the study, which was approved by the Regional Ethical Review Board at Lund University, Sweden (Decision numbers: 1999-99; 2005-471 and 2010-392).

Acknowledgments

Preliminary results from this study were presented by Per-Olof Östergren at the 12th European Public Health Conference in Stockholm, 1-4 November 2017. The authors would like to thank Elizabeth Cantor-Graae for language consulting.

For peer review only

For peer review only - http://bmjopen.bmj.com/site/about/guidelines.xhtml

References

1 Alleweldt F, Kara S, Graham R, et. al. (2013). The over-indebtedness of European households: updated mapping of the situation, nature and causes, effects and initiatives for alleviating its impact – Part 1: Synthesis of findings. Retrieved from https://ec.europa.eu/info/sites/default/files/final-report-on-over-indebtedness-of-european-households-synthesis-of-findings_december2013_en.pdf. Date of access 28 January 2022.

2 OECD. (2017). Household debt (indicator) doi: 10.1787/f03b6469-en. Date of access 19 October 2017.

3 Roos J. Konsumtionsrapporten 2014. In. Edited by Archive GUPE: Centrum för konsumtionsvetenskap, Göteborg University; 2014: 54. Retrieved from: http://hdl.handle.net/2077/37778. Date of access 28 January 2022.

4 SOU 2013:72. Ut ur skuldfällan. Betänkande av 2012 års skuldsaneringsutredning. In.: Government offices of Sweden; 2013. Retrieved from: https://data.riksdagen.se/fil/A237B785-C16A-4E50-B6E1-C8CE2AD22442. Date of access 28 January 2022.

5 Simovart MA, Saare K, Sein K. Protection of Consumer Rights in SMS Loan Agreements', *European Review of Private Law* 2010;1:129-42. Retrieved from: https://kluwerlawonline.com/journalarticle/European+Review+of+Private+Law/18.1/ERPL2010006. Date of access 28 January 2022.

6 Ahlström R. Bilaga 1: Överskuldsättningens kostnader. In: *Riksrevisionen granskar.* Riksrevisionen; 2015. Retrieved from: https://www.riksrevisionen.se/download/18.78ae827d1605526e94b2db65/1518435497252/Bilaga1.pdf. Date of access 28 January 2022.

For peer review only - http://bmjopen.bmj.com/site/about/guidelines.xhtml

7 Oyesanya M, Lopez-Morinigo J, Dutta R. Systematic review of suicide in economic recession. *World J Psychiatry* 2015;5:243-54.

8 Frasquilho D, Matos MG, Salonna F, et al. Mental health outcomes in times of economic recession: a systematic literature review. *BMC Public Health* 2016;16:115.

9 Ferreira MB, de Almeida F, Soro JC, et al. On the Relation Between Over-Indebtedness and Well-Being: An Analysis of the Mechanisms Influencing Health, Sleep, Life Satisfaction, and Emotional Well-Being. *Front Psychol* 2021;12:591875. https://doi.org/10.3389/fpsyg.2021.591875

10 Warth J, Beckmann N, Puth MT, et al. Association between over-indebtedness and antidepressant use: A cross-sectional analysis. *Plos One* 2020;15:e0236393.

11 Bialowolski P, Weziak-Bialowolska D, VanderWeele TJ. The impact of savings and credit on health and health behaviours: an outcome-wide longitudinal approach. *Int J Public Health* 2019; 64:573-84.

12 Turunen E, Hiilamo H. Health effects of indebtedness: a systematic review. *BMC Public Health* 2014;14:489.

13 Selenko E, Batinic B. Beyond debt. A moderator analysis of the relationship between perceived financial strain and mental health. *Soc Sci Med* 2011;73:1725-32.

14 Sweet E, Nandi A, Adam EK et al. The high price of debt: household financial debt and its impact on mental and physical health. *Soc Sci Med* 2013;91:94-100.

15 Blomgren J, Maunula N, Hiilamo H. Over-indebtedness and chronic disease: a linked register-based study of Finnish men and women during 1995-2010. *Int J Public Health* 2016;61:535-44.

For peer review only - http://bmjopen.bmj.com/site/about/guidelines.xhtml

16 Hagquist CEI. Economic stress and perceived health among adolescents in Sweden. *J Adolesc Health* 1998;22:250-7.

17 Richardson T, Elliott P, Roberts R. The relationship between personal unsecured debt and mental and physical health: a systematic review and meta-analysis. *Clin Psychol Rev* 2013;33:1148-62.

18 Davies W, Montgomerie J, Wallin S. Financial melancholia: Financial health and indebtedness; 2015. Retrieved at: https://research.gold.ac.uk/id/eprint/16269/1/FinancialMelancholiaMentalHealthandIndebtedness.pdf. Date of access 28 January 2022.

19 Levinsson H, Jiborn M. Minuskontot: Ekonomiska villkor för personer med psykisk funktionsnedsättning. In.: Hjärnkoll/Nationell samverkan för Psykisk Hälsa; 2013. Retrieved from: https://lucris.lub.lu.se/ws/portalfiles/portal/6002117/4864878.pdf. Date of access 28 January 2022.

20 Carlsson F, Merlo J, Lindström M, et al. Representativity of a postal public health questionnaire survey in Sweden, with special reference to ethnic differences in participation. *Scand J Public Health* 2006;34:132-9.

21 Canivet C, Nilsson A, Björk J, et al. Assessment of selection bias due to dropouts in the follow-up of the Scania Public Health Cohort. *Scand J Public Health* 2021;49:457-64.

22 Goldberg DP, Gater R, Sartorius N, et al. The validity of two versions of the GHQ in the WHO study of mental illness in general health care. *Psychol Med* 1997;27:191-7.

23 Goldberg DP, Oldehinkel T, Ormel J. Why GHQ threshold varies from one place to another. *Psychol Med* 1998;28:915-21.

For peer review only

24 Ahnquist J, Wamala SP: Economic hardships in adulthood and mental health in Sweden. The Swedish National Public Health Survey 2009. *BMC Public Health* 2011;11:788. doi: 10.1186/1471-2458-11-788.

25 Canivet C, Bodin T, Emmelin M, et al. Precarious employment is a risk factor for poor mental health in young individuals in Sweden: a cohort study with multiple follow-ups. *BMC Public Health* 2016;16:687. doi: 10.1186/s12889-016-3358-5

26 Canivet C, Aronsson G, Bernhard-Oettel C, et al. The negative effects on mental health of being in a non-desired occupation in an increasingly precarious labour market. *SSM - Population Health* 2017; 3:516-24. https://doi.org/10.1016/j.ssmph.2017.05.009

27 Zou G. A modified poisson regression approach to prospective studies with binary data. *Am J Epidemiol* 2004;159:702-6.

28 Rothman K. Modern epidemiology. Boston: Little, Brown & Co 1986.

29 Diderichsen F, Hallqvist J, Whitehead M. Differential vulnerability and susceptibility: how to make use of recent development in our understanding of mediation and interaction to tackle health inequalities. *Int J Epidemiol* 2019;48:268-74.

30 Rosvall M, Grahn M, Modén B, et al. Hälsoförhållanden i Skåne: Folkhälsoenkät Skåne 2008: Universitetssjukhuset MAS; 2009. Retrieved from: https://utveckling.skane.se/siteassets/publikationer_dokument/folkhalsoenkat_skane_2008.pdf. Date of access 28 January 2022.

31 Nilsson A, Bonander C, Strömberg U, et al. Reweighting a Swedish health questionnaire survey using extensive population register and self-reported data for assessing and improving the validity of longitudinal associations. *PloS One* 2021;16:e0253969.

For peer review only

32 Hedborg A. Överskuldsättning i kreditsamhället? SOU 2013:78. Retrieved from: https://www.regeringen.se/49bb85/contentassets/7d6ed04741ab4b9badd089ed01fd3039/overskuldsattning-i-kreditsamhallet-sou-201378-hela-betankandet. Date of access 28 January 2022.

33 Lundin A, Hallgren M, Theobald H, et al. Validity of the 12-item version of the General Health Questionnaire in detecting depression in the general population. *Public Health* 2016:136:66-74.

34 Lundin A, Åhs J, Åsbring N, et al. Discriminant validity of the 12-item version of the general health questionnaire in a Swedish case-control study. *Nord J Psychiatry* 2017;71:171-9.

For peer review only

Reporting checklist for cohort study.

Based on the STROBE cohort guidelines.

Instructions to authors

Complete this checklist by entering the page numbers from your manuscript where readers will find each of the items listed below.

Your article may not currently address all the items on the checklist. Please modify your text to include the missing information. If you are certain that an item does not apply, please write "n/a" and provide a short explanation.

Upload your completed checklist as an extra file when you submit to a journal.

In your methods section, say that you used the STROBE cohortreporting guidelines, and cite them as:

von Elm E, Altman DG, Egger M, Pocock SJ, Gotzsche PC, Vandenbroucke JP. The Strengthening the Reporting of Observational Studies in Epidemiology (STROBE) Statement: guidelines for reporting observational studies.

		Reporting Item	Page Number
**Title and abstract**			
Title	#1a	Indicate the study's design with a commonly used term in the title or the abstract	3
Abstract	#1b	Provide in the abstract an informative and balanced summary of what was done and what was found	3
**Introduction**			
Background / rationale	#2	Explain the scientific background and rationale for the investigation being reported	5-7
Objectives	#3	State specific objectives, including any prespecified hypotheses	7
**Methods**			
Study design	#4	Present key elements of study design early in the paper	8-9

For peer review only

Setting	#5	Describe the setting, locations, and relevant dates, including periods of recruitment, exposure, follow-up, and data collection	8-9
Eligibility criteria	#6a	Give the eligibility criteria, and the sources and methods of selection of participants. Describe methods of follow-up.	8-9
Eligibility criteria	#6b	For matched studies, give matching criteria and number of exposed and unexposed	n/a
Variables	#7	Clearly define all outcomes, exposures, predictors, potential confounders, and effect modifiers. Give diagnostic criteria, if applicable	9-10
Data sources / measurement	#8	For each variable of interest give sources of data and details of methods of assessment (measurement). Describe comparability of assessment methods if there is more than one group. Give information separately for for exposed and unexposed groups if applicable.	9-10
Bias	#9	Describe any efforts to address potential sources of bias	10-11
Study size	#10	Explain how the study size was arrived at	8-9
Quantitative variables	#11	Explain how quantitative variables were handled in the analyses. If applicable, describe which groupings were chosen, and why	9-10
Statistical methods	#12a	Describe all statistical methods, including those used to control for confounding	
10-11			
Statistical methods	#12b	Describe any methods used to examine subgroups and interactions	10-11
Statistical methods	#12c	Explain how missing data were addressed	8-9
Statistical methods	#12d	If applicable, explain how loss to follow-up was addressed	8-9
Statistical methods	#12e	Describe any sensitivity analyses	
n/a			

Results

For peer review only

Participants	#13a	Report numbers of individuals at each stage of study—eg numbers potentially eligible, examined for eligibility, confirmed eligible, included in the study, completing follow-up, and analysed. Give information separately for for exposed and unexposed groups if applicable.	11 and table 1
Participants	#13b	Give reasons for non-participation at each stage	8-9
Participants	#13c	Consider use of a flow diagram	n/a
Descriptive data	#14a	Give characteristics of study participants (eg demographic, clinical, social) and information on exposures and potential confounders. Give information separately for exposed and unexposed groups if applicable.	11 table 1
Descriptive data	#14b	Indicate number of participants with missing data for each variable of interest	table 1
Descriptive data	#14c	Summarise follow-up time (eg, average and total amount)	8
Outcome data	#15	Report numbers of outcome events or summary measures over time. Give information separately for exposed and unexposed groups if applicable.	11-12 table 2
Main results	#16a	Give unadjusted estimates and, if applicable, confounder-adjusted estimates and their precision (eg, 95% confidence interval). Make clear which confounders were adjusted for and why they were included	11-13 table 2
Main results	#16b	Report category boundaries when continuous variables were categorized	9-10
Main results	#16c	If relevant, consider translating estimates of relative risk into absolute risk for a meaningful time period	n/a

For peer review only

Other analyses	#17	Report other analyses done—eg analyses of subgroups and interactions, and sensitivity analyses	12-13 tables 4a-b13
**Discussion**			
Key results	#18	Summarise key results with reference to study objectives	13
Limitations	#19	Discuss limitations of the study, taking into account sources of potential bias or imprecision. Discuss both direction and magnitude of any potential bias.	15-16
Interpretation	#20	Give a cautious overall interpretation considering objectives, limitations, multiplicity of analyses, results from similar studies, and other relevant evidence.	13-15
Generalisability	#21	Discuss the generalisability (external validity) of the study results	13-15, 17
**Other Information**			
Funding	#22	Give the source of funding and the role of the funders for the present study and, if applicable, for the original study on which the present article is based	17

Notes:

- 13a: 11 and table 1
- 15: 11-12 table 2
- 16a: 11-13 table 2
- 17: 12-13 tables 4a-b13 The STROBE checklist is distributed under the terms of the Creative Commons Attribution License CC-BY. This checklist was completed on 05. February 2022 using https://www.goodreports.org/, a tool made by the EQUATOR Network in collaboration with Penelope.ai

For peer review only

Selling one's future: Over-indebtedness and the risk of poor mental health and the role of precarious employment – results from the Scania Public Health Cohort, Sweden

Journal:	*BMJ Open*
Manuscript ID	bmjopen-2022-061797.R2
Article Type:	Original research
Date Submitted by the Author:	19-Oct-2022
Complete List of Authors:	Östergren, Per-Olof; Lund University, Department of Clinical Sciences Malmö<br>Bodin, Theo; Karolinska Institutet; Center for Occupational and Environmental Medicine<br>Canivet, Catarina ; Lund University, Dept of Clinical Sciences Malmo<br>Moghaddassi, Mahnaz; Lund University, Department of Clinical Sciences Malmö<br>Vilhelmsson, Andreas; Lund University, Department of Laboratory Medicine
**Primary Subject Heading**:	Epidemiology
Secondary Subject Heading:	Mental health, Public health
Keywords:	MENTAL HEALTH, EPIDEMIOLOGY, Health policy < HEALTH SERVICES ADMINISTRATION & MANAGEMENT, PUBLIC HEALTH, Public health < INFECTIOUS DISEASES

SCHOLARONE™ Manuscripts

I, the Submitting Author has the right to grant and does grant on behalf of all authors of the Work (as defined in the below author licence), an exclusive licence and/or a non-exclusive licence for contributions from authors who are: i) UK Crown employees; ii) where BMJ has agreed a CC-BY licence shall apply, and/or iii) in accordance with the terms applicable for US Federal Government officers or employees acting as part of their official duties; on a worldwide, perpetual, irrevocable, royalty-free basis to BMJ Publishing Group Ltd ("BMJ") its licensees and where the relevant Journal is co-owned by BMJ to the co-owners of the Journal, to publish the Work in this journal and any other BMJ products and to exploit all rights, as set out in our licence.

The Submitting Author accepts and understands that any supply made under these terms is made by BMJ to the Submitting Author unless you are acting as an employee on behalf of your employer or a postgraduate student of an affiliated institution which is paying any applicable article publishing charge ("APC") for Open Access articles. Where the Submitting Author wishes to make the Work available on an Open Access basis (and intends to pay the relevant APC), the terms of reuse of such Open Access shall be governed by a Creative Commons licence – details of these licences and which Creative Commons licence will apply to this Work are set out in our licence referred to above.

Other than as permitted in any relevant BMJ Author's Self Archiving Policies, I confirm this Work has not been accepted for publication elsewhere, is not being considered for publication elsewhere and does not duplicate material already published. I confirm all authors consent to publication of this Work and authorise the granting of this licence.

For peer review only

Selling one's future: Over-indebtedness and the risk of poor mental health and the role of precarious employment – results from the Scania Public Health Cohort, Sweden

Per-Olof Östergren[1], Theo Bodin[2,3], Catarina Canivet[1], Mahnaz Moghaddassi[1], Andreas Vilhelmsson[4]

1 Social Medicine and Global Health, Department of Clinical Sciences Malmö, Lund University, Lund, Sweden

2 Unit of Occupational Medicine, Institute of Environmental Medicine, Karolinska Institutet, Stockholm, Sweden

3 Center for Occupational and Environmental Medicine, Stockholm Region, Stockholm, Sweden

4 Division of Occupational and Environmental Medicine, Department of Laboratory Medicine, Lund University, Lund, Sweden

Corresponding author: Per-Olof Östergren, per-olof.ostergren@med.lu.se, +46 705160092

Word count abstract: 300

Word count article: 4224

For peer review only - http://bmjopen.bmj.com/site/about/guidelines.xhtml

Abstract

OBJECTIVES: The credit market has expanded rapidly, increasing the risk of over-indebtedness among those who lack secure employment or adequate income, an issue of concern in the Covid-19 aftermath. We investigated the role of over-indebtedness for developing poor mental health, and whether this impact is modified by age, gender, educational level, or being in precarious employment.

METHODS: This is a cohort study using data from the Swedish Scania Public Health Cohort, based on individuals randomly selected from the general adult population in Scania, southern Sweden, initiated in 1999/2000 (response rate 58%) with follow-ups in 2005 and 2010. Over-indebtedness was assessed by combining information on cash margin and difficulty in paying household bills. Mental health was assessed by General Health Questionnaire-12. Those with poor mental health at baseline were excluded, and the analyses were further restricted to vocationally active individuals with complete data on main variables, resulting in 1256 men and 1539 women.

RESULTS: Over-indebtedness was more common among women, among persons with a low educational level, born abroad, and with a precarious employment at baseline. The age-adjusted incidence rate ratio (IRR) for poor mental health in 2010 among individuals exposed to over-indebtedness in 1999/2000 or 2005 was 2.2 (95% CI: 1.7–2.8). Adjusting for educational level, country of origin, and precarious employment in 1999/2000 or 2005, yielded an IRR of 2.0; 95% CI: 1.6–2.6. An interaction analysis indicated that a high level of education may act synergistically with over-indebtedness, regarding poor mental health among men.

CONCLUSIONS: Over-indebtedness was related to unfavourable societal power relations, regarding social class, gender, and foreign birth. Precarious employment was independently linked to poor mental health and may also mediate the effect by over-indebtedness. The Covid-19 pandemic might entail increased over-indebtedness, which should be acknowledged in policies aiming at buffering social effects of the pandemic.

For peer review only

For peer review only

For peer review only - http://bmjopen.bmj.com/site/about/guidelines.xhtml

Strengths and limitations of this study

- This was a prospective cohort study with 2795 vocationally active men and women randomly selected from the general adult population.
- The exposure and outcome variables have been used and validated in previous studies.
- Detailed information concerning employment history and details regarding current empolyment for each individual was used for determining precarious empolyment status.
- Non-response and missing data among respondents may have caused some selection bias.
- We lacked access to an objective measure regarding over-indebtedness, which may have caused misclassification.

For peer review only

For peer review only - http://bmjopen.bmj.com/site/about/guidelines.xhtml

Introduction

Credit plays a paramount role in modern economies, since it increases the possibility to make investments that can generate future financial and other gains and to allocate lifetime income in a more optimal relation to need. From a health perspective, such resources are very likely related to opportunities for improved health over the life-course. However, when the credit market targets those who cannot make ends meet, this can cause increasing indebtedness, which can lead to poorer health, both due to loss of material and other resources and to exposure to growing stress and worries about one´s loss of control over the household economy. Thus, the proportion of individuals who are prone to over-indebtedness will most likely expand considerably in the aftermath of the Covid-19 pandemic. Moreover, indebtedness could be expected to have a differential effect on health, much depending on factors such as socioeconomic position, country of birth (i.e. migration background), and age.

The credit market has expanded over the last decades leading to a build-up of consumer debt. However, it was not until the financial crisis in 2007-2008 that an increased proportion of households that had difficulties making ends meet was noted in the EU.[1] As of 2016, the household debt in relation to disposable income was more than twice as high in the OECD compared to 1996.[2] Measured as share of GDP, the indebtedness of Swedish households has doubled since the 1990s.[3] According to public reports, the number of over-indebted individuals in Sweden amounts to between 6 and 18 per cent of the population, depending on definition. The subsequent yearly societal cost is estimated to 30 billion SEK.[4] The credit market has also changed in terms of type of credit providers and types of 'products', e.g. so called 'pay-day loans' as a temporary financial solution when financial means are insufficient to cover daily cost of living because of inadequate income and lack of savings.[5] These types of loans have at the same time become very accessible via the Internet and mobile phones. Since the interest rates and fees generally are very high for these loans, this could

For peer review only - http://bmjopen.bmj.com/site/about/guidelines.xhtml

quickly lead to a spiral of indebtedness where the loan-taker in practice ends up owing the lender a substantial proportion of his/her future income.[6]

Over-indebtedness can be defined as a situation, persisting for a sustained period of time, in which daily cost of living exceeds income, and in which the imbalance cannot be compensated by savings. This will inevitably lead to use of credit in one way or another, the cost of which will add to the discrepancy between expenditure on the one side and income and savings on the other. This situation could represent an emerging significant stressor and determinant of ill health, and thereby constitute a source of health inequality that is likely to be overlooked in research on income or wealth and health, since information concerning over-indebtedness can not easily be retrieved from registers on income, wealth, or social transfers.

A growing number of scientific studies on the indirect and direct association between over-indebtedness and health have been published in the scientific literature. Systematic reviews have provided support for the assumption that the financial recession has been associated with an increase in suicide rates in high-income countries such as Europe and North America.[7] Further, socioeconomic mediators such as unemployment, income decline, and unmanageable debts are significantly associated with poor mental wellbeing, increased rates of common mental disorders, substance-related disorders, and suicidal behaviours.[8-11] Individuals who cannot make their loan payments have been found to suffer from depression and have suicidal ideation more often compared to those without such problems.[12] Another study investigating predictors of mental health in people on the verge of bankruptcy found that perceived financial strain was an important predictor of poor mental health.[13] However, most of the studies have been cross-sectional, and more longitudinal studies are needed to corroborate the mentioned findings.

High debt relative to available assets has also been associated with poorer self-assessed general health, higher diastolic blood pressure, obesity, poorer health-related behaviour, and myocardial

For peer review only

For peer review only - http://bmjopen.bmj.com/site/about/guidelines.xhtml

infarction.[14] The effect of over-indebtedness on health has been found to be stronger for women than for men.[15] In a life-course perspective, poor self-rated health has been found to be even more prevalent among those who have experienced economic stress both early in life and later on.[16]

According to a systematic review and meta-analysis examining the relationship between personal debt and mental health,[17] ten epidemiological studies with nationally representative samples of the general population were found, whereof seven came from the UK and nine out of ten were cross-sectional. Unsurprisingly, very high odds ratios for different mental health outcomes were found. In many cases the causality pattern could be described as a vicious circle, which could start either by a decrease in income or in health. Poor mental health provoked by over-indebtedness tends to increase the risk of further financial problems and so forth.[18] Moreover, over-indebtedness or coping with excessive credit is associated with subjective distress such as shame, anxiety, and apathy.[19] The mentioned process could involve mediating mechanisms, as well as situations where certain exposures modify the effect of each other, such that already vulnerable groups, e.g. individuals with low education or in a precarious employment situation, are hit more severely by over-indebtedness in terms of resulting poor mental health.

To the best of our knowledge, evidence regarding the role of precarious employment as a mediator or modifier of the impact of over-indebtedness on mental health is lacking.

In summary, previous research relies heavily on cross-sectional data from the UK, why studies from other national contexts are needed. Moreover, because of the plausible bidirectional causal pathways, prospective studies of long duration with several assessments of both types of variables, over-indebtedness as well as mental health, are preferable. Also, as mentioned above, little is known about the role of precarious employment and the differential health effects across sociodemographic groups.

For peer review only - http://bmjopen.bmj.com/site/about/guidelines.xhtml

Objectives

The aim of the study was to investigate the role of over-indebtedness for the development of poor mental health in a longitudinal perspective, and whether this impact is modified by age, gender, educational level, housing tenure, or being in a precarious employment situation.

Methods

Study population

The present cohort was established in 1999/2000 and followed up in 2005 and 2010.[20] At baseline, a postal questionnaire was sent out to 25 000 men and women, 18–80 years old. These individuals were randomly selected from the population register, such that equal representation was achieved from all municipalities in the county of Scania (population 1.3 million), Sweden. In total, 13589 out of 23437 eligible individuals returned a completed questionnaire (response rate 58%). All of those who responded at baseline and still residing in the county were invited to follow-up after five and ten years. Out of 12002 respondents alive and still living in the region after 10 years, there were 8206, i.e. 68%, who also participated in the 2005 and 2010 inquiries.

A comparison between these 8206 respondents and the corresponding Scania general population at baseline [20] showed that younger individuals, men, and individuals with a low educational level were slightly underrepresented among respondents. However, a recently published study from this cohort, showed very little proof of selection bias regarding common health outcomes, includung mental health.[21]

For peer review only

Since one aim of the study was to look into the relationship between precarious employment and over-indebtedness and mental health, we firstly excluded individuals who were not vocationally active, i.e. at baseline were retired, on disability pension, or on long-term sick leave, and also those who answered that they did not wish to work a year from now (N=1744). Out of the remaining 6462 persons, those with lacking data on employment precariousness (N=2479) were excluded; out of these, 1106 were 55–80 years old at baseline, i.e. were qualified for old-age retirement during the studied window of time. Further, we excluded individuals who lacked baseline data regarding the variables included in the multivariable analyses (rather than assigning them as 'internal' missing); i.e. housing tenure (N=159) or educational level (N=158), also those lacking baseline and 2005 information on over-indebtedness (N=249), furthermore those who lacked information regarding mental health at all follow-ups (N=161). Lastly, in order to reduce the influence of reverse causation, we also excluded individuals with poor mental health at baseline (N=1563), instead of controlling for this in the multivariable analysis. Thus, the final study population consisted of 2795 individuals, 1256 men and 1539 women.

Outcome variable – poor mental health

Mental health was measured at all three time points of assessment, using the 12-item version of the General Health Questionnaire (GHQ-12). We used the 0-0-1-1 scoring method (range 0–12) recommended by the creators of the instrument, [22] with poor mental health or 'GHQ-caseness' determined by the population mean and defined as a score of 2 or higher.[23]

Over-indebtedness

Two items have been used previously in several Swedish studies to evaluate economic hardship, namely inability to meet expenses and lacking cash reserves.[24] For the former we used the question 'How often during the last 12 months have you had problems paying your bills?' and for the latter, 'In

For peer review only - http://bmjopen.bmj.com/site/about/guidelines.xhtml

case of an unforeseen emergency, do you have the capacity to raise 12000 SEK *(approximately 1100 EUR)* in a week's time?' The same questions were asked in 1999/2000, 2005, and 2010, but the specified amount was raised to 14000 SEK, approximately 1300 EUR, in the 2010 survey. Over-indebtedness was defined as affirming 'about half of the months' (or more often) on the first question and answering 'no' on the second.

Other variables

Age was classified into four groups, except in the interaction analysis, where it was dichotomised, and in the multivariate analysis, where it was used as a continuous variable. Country of origin was recorded as 'born in Sweden' (yes/no). Educational level at baseline was determined by the self-reported total years of formal education and classified into four groups. Housing tenure was dichotomised as either owned or rented residence. For employment precariousness, we used a dichotomous variable, based on a combination of data on present unemployment, episode of involuntary unemployment during the past three years (no/yes), currently temporarily vs. permanently employed, and perceived job insecurity.[25, 26]

Statistical methods

The relationships between background factors and poor mental health in 2010 are presented as percentages and age-adjusted incidence rate ratios (IRR), which are a good estimate of relative risks, using a modified Poisson regression model with robust standard errors.[27] In the multivariate analysis, over-indebtedness was tested against the outcome with the stepwise addition of educational level, country of origin, and precarious employment.

The tests for effect modification were performed with simple dummy variables. The synergy indices were calculated as proposed by Rothman.[28] according to which a synergy index > 1 may indicate a

For peer review only

synergistic effect, and a synergy index < 1 an antagonistic effect between two exposure variables. In the interaction analysis, educational level was dichotomised as '12 years or less' versus '13 years or more'. There was a negative relationship between educational level and over-indebtedness (see Results), while the relationship with the outcome variable poor mental health at follow-up was positive (see Results), and therefore a high educational level was designated as exposure.

Two standard statistical analysis programmes were used, IBM SPSS Statistics for Windows, Version 22.0, Armonk, NY: IBM Corp. and Stata Statistical Software: Release 12, College Station, TX: StataCorp LP.

Patient and public involvement statement

Patient or public involvement in the design, reporting or dissemination of results was not possible.

Results

Table 1 shows the prevalence of over-indebtedness in the total study population at baseline and also separately by gender. Overall, the prevalence of overindebted individuals was 4.2%, but with a clear difference between men and women (2.8 versus 5.3%, respectively). The only clear age-related difference in over-indebtedness consisted of a low prevalence in participants aged 55 and above. Over-indebtedness was more common in those with an educational level of 10–12 years, vs. those with less or with more years. When educational level was dichotomised as '12 years or less' vs. '13 years or more', 5.5% of those with a low educational level were overindebted at baseline, vs. 2.7% of those with a high educational level (data not shown in tables). Over-indebtedness was three times as common among those born in another country than Sweden. As perhaps expected, over-indebtedness was almost four times as prevalent among those who reported a precarious employment situation and

For peer review only - http://bmjopen.bmj.com/site/about/guidelines.xhtml

among those who rented their dwelling (compared to those who owned it). The gender pattern was largely similar for all factors.

For peer review only - http://bmjopen.bmj.com/site/about/guidelines.xhtml

Table 1

Background characteristics at baseline in 1999/2000 of 1256 men and 1539 women from the Scania Public Health Cohort, in relation to over-indebtedness.

		All				Men				Women			
		Total	No	Yes[a]	% Yes	Total	No	Yes	% Yes	Total	No	Yes	% Yes
Total		2795	2679	116	4.2	1256	1221	35	2.8	1539	1458	81	5.3
Age	18–24	207	196	11	5.3	96	92	4	4.2	111	104	7	6.3
	25–34	621	596	25	4.0	278	272	6	2.2	343	324	19	5.5
	35–44	750	710	40	5.3	324	314	10	3.1	426	396	30	7.0
	45–54	966	930	36	3.7	430	416	14	3.3	536	514	22	4.1
	55–63	251	247	4	1.6	128	127	1	0.8	123	120	3	2.4
Educational level	9 y or less	502	484	18	3.6	265	256	9	3.4	237	228	9	3.8
	10–12	954	892	62	6.5	437	416	21	4.8	517	476	41	7.9
	13–14	633	609	24	3.8	253	250	3	1.2	380	359	21	5.5
	15 y or more	706	694	12	1.7	301	299	2	0.7	405	395	10	2.5
Born in Sweden	Yes	2588	2494	94	3.6	1163	1136	27	2.3	1425	1358	67	4.7
	No	207	185	22	10.6	93	85	8	8.6	114	100	14	12.3
Precarious employment	No	2051	2004	47	2.3	943	926	17	1.8	1108	1078	30	2.7
	Yes	744	675	69	9.3	313	295	18	5.8	431	380	51	11.8
Housing tenure	Owned	2176	2112	64	2.9	990	969	21	2.1	1186	1143	43	3.6
	Rented	619	567	52	8.4	266	252	14	5.3	353	315	38	10.8

[a] Over-indebted at baseline

For peer review only

For peer review only - http://bmjopen.bmj.com/site/about/guidelines.xhtml

Table 2 shows the results of the longitudinal analysis, where poor mental health at follow-up in 2010 was used as the outcome. The age-adjusted risk for poor mental health at follow-up in overindebted individuals, measured as incidence rate ratio (IRR), was 2.2 (95% CI: 1.7–2.8) in the entire group, 2.6 (1.8–3.9) for men, and 1.9 (1.4–2.6) for women. The risk for poor mental health was four times higher in those who were 18–24 years at baseline, with an IRR of 4.1 (2.3–7.4), and decreased successively with increasing age, compared to those who were 55 or older at baseline, which was used as the reference group. That pattern was similar in men and women. Those with an educational level of 13–14 years at baseline had a slightly higher risk of poor mental health at follow-up (IRR=1.4; 95% CI: 1.01–2.0) than those with 9 years or less, which was used as the reference. When educational level was dichotomised, 15.1% of those with a high educational level had poor mental health at follow-up vs. 13.9% of those with a low educational level (data not shown in tables). Foreign birth was related to poor mental health in men only, with an IRR of 1.6 (1.01–2.6). Having a precarious employment situation during the period 1999/2000 to 2005 was statistically significantly related to poor mental health in 2010 (IRR= 1.4; 95% CI:1.2–1.7), albeit also here with a gender difference; the IRR for men was 1.8 (1.3–2.4) and for women 1.2 (0.95–1.5). Housing tenure (rented dwelling) at baseline was not related to poor mental health in 2010.

For peer review only

Table 2
Background characteristics from 1999/2000 through 2005 in relation to poor mental health in 2010.

		All				Men				Women			
		N cases poor mental health [a]	% cases poor mental health	IRR [b]	95% CI [c]	N cases poor mental health	% cases poor mental health	IRR	95% CI	N cases poor mental health	% cases poor mental health	IRR	95% CI
Total		404	14.5			144	11.5			260	16.9		
Over-indebtedness in 1999/2000 or 2005, at least once	No	348	13.3	1		124	10.4	1		224	15.7		1
	Yes	56	31.8	2.2	1.7–2.8	20	31.3	2.6	1.8–3.9	36	32.1	1.9	1.4–2.6
Age in 1999/2000	18–24	44	21.3	4.1	2.3–7.4	17	17.7	5.7	2.0–16.3	27	24.3	3.3	1.6–6.8
	25–34	125	20.1	3.9	2.2–6.8	46	16.5	5.3	2.0–14.4	79	23.0	3.1	1.6–6.1
	35–44	114	15.2	2.9	1.7–5.1	41	12.7	4.0	1.5–11.1	73	17.1	2.3	1.2–4.5
	45–54	108	11.2	2.2	1.2–3.8	36	8.4	2.7	0.97–7.4	72	13.4	1.8	0.9–3.6
	55–63	13	5.2	1		4	3.1	1		9	7.3	1	
Educational level in 1999/2000	9 y or less	47	9.4	1		18	6.8	1		29	12.2	1	
	10–12	155	16.2	1.3	0.9–1.8	61	14.0	1.4	0.8–2.5	94	18.2	1.1	0.8–1.7
	13–14	107	16.9	1.4	1.01–2.0	27	10.7	1.2	0.7–2.1	80	21.1	1.4	0.9–2.1
	15 y or more	95	13.5	1.2	0.8–1.6	38	12.6	1.4	0.8–2.5	57	14.1	0.9	0.6–1.4
Born in Sweden	Yes	369	14.3	1		128	11.0	1		241	16.9	1	
	No	35	16.9	1.2	0.9–1.6	16	17.2	1.6	1.01–2.6	19	16.7	1.0	0.6–1.5
Precarious employment in 1999/2000 or 2005, at least once	No	211	12.0	1		69	8.5	1		142	15.0	1	
	Yes	193	18.6	1.4	1.2–1.7	75	16.7	1.8	1.3–2.4	118	20.0	1.2	0.95–1.5
Housing tenure in 1999/2000	Owned	292	13.4	1		101	10.2	1		191	16.1	1	
	Rented	112	18.1	1.1	0.9–1.4	43	16.2	1.3	0.9–1.9	69	19.5	1.0	0.8–1.3

[a] Poor mental health is defined as GHQ-caseness; [b] IRR, incidence rate ratio, age-adjusted; [c] CI, confidence interval

For peer review only

For peer review only - http://bmjopen.bmj.com/site/about/guidelines.xhtml

Table 3 shows the effect on the risk estimate for poor mental health at follow-up when exposed to over-indebtedness during the period 1999/2000 to 2005, with a step-wise introduction of the main covariates. In the final step, when precarious employment situation during 1999/2000 to 2005 was introduced into the analysis, the risk estimate for poor mental health regarding exposure to over-indebtedness 1999/2000 to 2005 decreased about 10% for the entire group. The decrease in IRR in the fully adjusted model was slightly larger for men than for women. As a general remark, we find it possible that this is a case of over-adjustment, since all the introduced covariates could be included in the same causal chains, as well as in separate ones, and we have no way to disentangle this.

For peer review only

Table 3

Over-indebtedness at least once in 1999/2000 or 2005 in relation to the outcome poor mental health at follow-up in 2010, with forward stepwise addition of potential confounders; educational level in 1999/2000, country of origin, and precarious employment at least once in 1999/2000 or 2005.

			Model 1 Age-adjusted		Model 2 Model 1 + adjusted for educational level in 1999/2000		Model 3 Model 2 + adjusted for country of origin		Model 4 Model 3 + adjusted for precarious employment in 1999/2000 or 2005	
			IRR[a]	95% CI[b]	IRR	95% CI	IRR	95% CI	IRR	95% CI
All	Over-indebtedness	Yes versus no	2.2	1.7–2.8	2.2	1.7–2.8	2.2	1.7–2.8	2.0	1.6–2.6
	Educational level	9 years or less			1		1		1	
		10–12 years			1.2	0.9–1.7	1.2	0.9–1.7	1.3	0.9–1.8
		13–14 years			1.4	1.03–2.0	1.4	1.03–2.0	1.5	1.1–2.1
		15 years or more			1.2	0.9–1.7	1.2	0.9–1.7	1.3	0.9–1.8
	Born in Sweden	No versus yes					1.1	0.8–1.4	1.0	0.7–1.4
	Precarious employment	Yes versus no							1.3	1.1–1.6
Men	Over-indebtedness	Yes versus no	2.6	1.8–3.9	2.7	1.8–4.2	2.6	1.7–4.0	2.2	1.4–3.5
	Educational level	9 years or less			1		1		1	
		10–12 years			1.4	0.8–2.4	1.4	0.8–2.4	1.4	0.8–2.4
		13–14 years			1.2	0.7–2.2	1.3	0.7–2.2	1.2	0.7–2.2
		15 years or more			1.6	0.9–2.8	1.6	0.9–2.7	1.6	0.9–2.8
	Born in Sweden	No versus yes					1.3	0.8–2.1	1.2	0.7–1.9
	Precarious employment	Yes versus no							1.6	1.2–2.3
Women	Over-indebtedness	Yes versus no	1.9	1.4–2.6	1.9	1.4–2.5	1.9	1.4–2.6	1.8	1.4–2.5
	Educational level	9 years or less			1		1		1	
		10–12 years			1.1	0.7–1.7	1.1	0.7–1.7	1.1	0.8–1.7
		13–14 years			1.4	0.9–2.1	1.4	0.9–2.1	1.4	0.9–2.1
		15 years or more			1.0	0.6–1.5	1.0	0.6–1.5	1.0	0.6–1.5
	Born in Sweden	No versus yes					0.9	0.6–1.4	0.9	0.6–1.3
	Precarious employment	Yes versus no							1.1	0.9–1.4

[a] IRR, incidence rate ratio; [b] CI, confidence interval

For peer review only - http://bmjopen.bmj.com/site/about/guidelines.xhtml

As it is of considerable interest to investigate whether there are certain segments of the population that are more vulnerable concerning the risk of developing poor mental health when exposed to over-indebtedness, we examined this concerning age and level of education. As seen in Table 4A, there was no indication of a synergistic effect of young age and over-indebtedness on the risk of poor mental health at follow-up. However, as seen in Table 4B, while there was likewise no interaction between a high educational level and over-indebtedness in women, a higher risk for poor mental health was seen in men with this particular combination of risk factors. The IRR for men was 3.8 (95% CI: 1.8–8.0), and the resulting synergy index 1.8.

For peer review only

For peer review only - http://bmjopen.bmj.com/site/about/guidelines.xhtml

Table 4A

Interaction analyses, by gender, with synergy indices regarding age group in 1999/2000 and over-indebtedness at least once in 1999/2000 or 2005, in relation to poor mental health in 2010.

	All					Men					Women				
Age group in 1999/2000 (18–34 vs 35–63) and over-indebtedness in 1999/2000 or 2005	N	% cases poor mental health	IRR[a]	95% CI[b]	SI[c]	N	% cases poor mental health	IRR[a]	95% CI[b]	SI[c]	N	% cases poor mental health	IRR[a]	95% CI[b]	SI[c]
Older & No over-indebtedness	1859	11.0	1			844	8.4	1			1015	13.2	1		
Older & Over-indebtedness	108	27.8	2.5	1.8–3.5		38	26.3	3.1	1.8–5.6		70	28.6	2.2	1.5–3.2	
Younger & No over-indebtedness	760	18.8	1.7	1.4–2.1		348	15.2	1.8	1.3–2.5		412	21.8	1.7	1.3–2.1	
Younger & Over-indebtedness	68	38.2	3.5	2.5–4.8	1.1	26	38.5	4.6	2.7–7.8	1.2	42	38.1	2.9	1.9–4.4	1.0
	2795					1256					1539				

[a] IRR, incidence rate ratio; [b] CI, confidence interval; [c] SI, synergy index

For peer review only - http://bmjopen.bmj.com/site/about/guidelines.xhtml

Table 4B

Interaction analyses, by gender, with synergy indices regarding educational level in 1999/2000 and over-indebtedness at least once in 1999/2000 or 2005, in relation to poor mental health in 2010.

	All					Men					Women				
Educational level in 1999/2000 and over-indebtedness in 1999/2000 or 2005	N	% cases poor mental health	IRR[a]	95% CI[b]	SI[c]	N	% cases poor mental health	IRR[a]	95% CI[b]	SI[c]	N	% cases poor mental health	IRR[a]	95% CI[b]	SI[c]
Low & No over-indebtedness	1336	12.4	1			649	9.9	1			687	14.8	1		
Low & Over-indebtedness	120	30.0	2.2	1.6–3.0		53	28.3	2.5	1.5–4.1		67	31.3	2.0	1.3–2.9	
High & No over-indebtedness	1283	14.2	1.1	0.9–1.3		543	11.0	1.1	0.8–1.5		740	16.5	1.1	0.9–1.4	
High & Over-indebtedness	56	35.7	2.6	1.8–3.8	1.2	11	45.5	3.8	1.8–8.0	1.8	45	33.3	2.1	1.3–3.2	1.0
	2795					1256					1539				

[a] IRR, age-adjusted incidence rate ratio; [b] CI, confidence interval; [c] SI, synergy index

For peer review only

Discussion

This study confirmed that vocationally active individuals who are exposed to over-indebtedness have an increased risk of developing poor mental health. Age-adjusted estimates showed that the risk was almost tripled for men and doubled for women in this general adult population sample of vocationally active individuals (18–65 years). Considering that about 4 % of all individuals in the cohort were exposed to over-indebtedness at baseline and probably substantially more during the follow-up period, over-indebtedness should be regarded as an important general population risk factor for poor mental health. This is pertinent especially in the aftermath of the Covid-19 pandemic, during which the number of vulnerable individuals in the adult population is likely to increase considerably.

At the same time, especially from a health equity perspective, it must be acknowledged that the prevalence and to some extent the impact of exposure to over-indebtedness in the population depend on sociodemographic circumstances. Previous reseaerch provides few examples of how this could be integrated into a coherent theoretical framework regarding health equity. We speculate that the reason that younger individuals are more exposed to over-indebtedness than middle-aged or older ones may partly have a 'life-course' explanation (mortgage loans are taken early in life and paid off over time), but there is also a generational effect due to the expansion of credit markets over time. For the other group disparities in over-indebtedness, reasons must be sought elsewhere. Women were more exposed than men, foreign-born more than those born in Sweden, and those with a low educational level more than those with a high level. In short, it seems that the risk for over-indebtedness follows all the main societal power structures, i.e. gender, social class, and ethnicity. Therefore, it is an important factor to consider when formulating and implementing policies for promoting health equity.

Regarding socioeconomic factors that logically should be strongly related to over-indebtedness, i.e. precarious employment and housing ownership (i.e. non-ownership), we found that both these factors were strongly related to over-indebtedness, but that only precarious employment was also related to the risk of developing poor mental

For peer review only - http://bmjopen.bmj.com/site/about/guidelines.xhtml

health during our observation period. However, when we performed an analysis to control for confounding from precarious employment, the impact of over-indebtedness on poor mental health was only moderately reduced. Considering a bidirectional relation between over-indebtedness and poor mental health, the choice to exclude individuals with poor mental health at baseline could have yielded an underestimation of the true association between the variables.

Health inequality could be the result of both differential exposure and differential impact when once exposed.[29] From the discussion above, it seems obvious that there is a clear case of differential exposure regarding over-indebtedness and sociodemographic factors. When we tested by means of two-way interactions whether we in addition could find any evidence for a differential impact, we concluded that this was not the case regarding age (Table 4A). We noted, however, that the combination of a high educational level and over-indebtedness (Table 4B) was associated with a synergy index of 1.0 in women, whereas the corresponding index in men was 1.8, thus indicating that these factors may act synergistically in a detrimental way for men. One could speculate that over-indebtedness may be especially hard to endure for a well-educated man, since over-indebtedness implies that one is financially not 'in control', whereas gender-based stereotypes of masculinity imply the opposite. Added to that might be the frustration caused by having invested many years in education and not being 'rewarded' by economic stability. Nevertheless, this finding relies on an analysis in which the number of exposed men is small, why the confidence intervals are large. Therefore, this result needs to be replicated in other research.

As shown in many studies as well as in this one, the basic power relations in society, among which would be those determined by gender, social class, and ethnicity, show a strong relation to the risk of becoming over-indebted. This is not surprising, because all the above mentioned power relations determine access to factors that could avoid a short- or long-term situation where credit is needed for securing stable living conditions. Not being able to do so will induce both a loss of control and a loss of material resources, situations that have been shown to increase individuals' susceptibility to poor health development, especially poor mental health. However, the

For peer review only

'Faustian deal' of using credit in this situation could make things worse, since a spiral of failing credit and declining mental health could place an individual in a situation where he or she in fact has sold a considerable part of future earnings to a moneylender institute. The possibility of having such debts cancelled in a regulated way, which is open to business enterprises through the legal bankruptcy framework, is very restricted for individuals in the Swedish context. This is particularly problematic in a health equity perspective because of the strong association between societal power structures and the risk of becoming overindebted, all of which could be magnified in the wake of the Covid-19 pandemic.

The results of this study are therefore very relevant for policy, not only regarding public health and health equity, but also for policy formulation and implementation in the judicial and social realms. A specific policy recommendation is to expand the possibilities to write off debt for the individual, in a similar way that now is possible for business enterprises, in order to prevent a vicious circle of poor mental health and increasing debt, which seems maleficent for all involved stakeholders. Because of the growing problem with over-indebtedness, it seems urgent to develop the information systems concerning individual's and household's financial situation, in a manner that high quality information becomes available, not only concerning income but equally concerning financial assets and debt. This, especially since increasing inequality appears to be better mirrored by the latter, rather than the former.

Strengths and weaknesses

This study utilises a prospective design using a large randomly selected general population sample of individuals in the occupationally active age-bracket in southern Sweden. A first selection took place in the very establishment of the Scania Public Health Cohort, since the response rate was 58 %.[30] This opens up for the introduction of selection bias. However, two studies that were specifically designed to elucidate selection bias in this cohort concluded that apart from an under-representation of foreign-born individuals, the responders were not statistically different from non-responders regarding other sociodemographic characteristics,[20] nor were

For peer review only

For peer review only - http://bmjopen.bmj.com/site/about/guidelines.xhtml

any signs of selection bias evident since the risk esimates for common illnesses (including mental health) were very similar between participants and drop outs.[21, 31] In the former study, it was also noted that health care utilisation did not differ between responders and non-responders during the baseline survey year. Moreover, such bias could in fact give rise to an underestimation, rather than an overestimation, of the observed differences, since it seems reasonable that heavily indebted individuals for several reasons (lack of postal address, etc.) could be underrepresented in the sample, especially if their mental health was poor.

The main exposure, over-indebtedness, was based on two questions that in combination have been deemed valid by other authors[24] to identify individuals with a financial situation indicating that their income for a longer period of time was insufficient to meet their regular household bills without having other financial assets (the definition of over-indebtedness). We believe that this measure might be more objective, since it builds on two factual circumstances, than measures solely based on a subjective assessment of financial stress. The prevalence of estimated over-indebtedness in our sample seems to be reasonably on par with the prevalence of over-indebtedness in the general Swedish population at the time of assessment.[32] A direct measure of over-indebtedness would be preferable, but this seems difficult to implement either by register studies or by surveys, since there is a whole range of credit forms to be covered, from regular bank loans to personal or even criminal types of credit that are very difficult to assess in a reliable and valid manner.

Our measure of precarious employment is based on relevant indicators and has been used in a recent study;[25] however, it has not been validated in relation to other existing instruments. Given that confounding from a precarious employment situation was rather weak, and considering that this factor very well could be involved in the same causal chain as the main exposure, we find it unlikely that the discussed possible misclassification had biased our finding to any degree of importance. Although we have assessed potential confounding from main socioeconomic background factors known to be linked to over-indebtedness, such as gender, educational level, age, and migration status, we cannot fully rule out residual confounding from other factors.

For peer review only - http://bmjopen.bmj.com/site/about/guidelines.xhtml

This study was based on self-reported data. However, the GHQ instrument has been extensively validated, and recent studies carried out in Sweden have shown that the GHQ-12 performs acceptably in detecting depression in the general population[33] and also performs acceptably in discriminating out-patients with depression and anxiety from healthy controls.[34]

Conclusions

This prospective study showed that exposure to over-indebtedness had a negative effect on vocationally active individual's mental health. It also showed that over-indebtedness is not uncommon in the general working population, and it therefore seems to be a significant contributor to poor mental health at the population level, especially since loss of employment and secure income is likely to increase considerably in the aftermath of Covid-19. Moreover, over-indebtedness was found to be clearly related to an unfavourable position with regard to societal power relations. The findings are therefore of importance not only for policy makers in the area of public health and health equity, but also for policy areas that determine the risk for becoming over-indebted, such as the social and judicial realms.

For peer review only

Funding

This work was funded by FORTE (Swedish Research Council for Health, Working Life and Welfare; Grant Number 2013-1269) and the Pufendorf Institute at Lund University (Credit Society Project). The funding institutions had no role in the design of the study, data collection, analysis and interpretation of data, or in writing of the manuscript.

Competing Interests

The authors declare that they have no conflict of interest.

For peer review only - http://bmjopen.bmj.com/site/about/guidelines.xhtml

Authors' Contributions

The study was concieved and designed by PÖ, CC and AV. The analysis was done by MM. TB drafted the first version of the manuscript. All authors took part in interpreting the results and writing subsequent drafts.

Data availability statement

All data could be obtained by request to the corresponding author.

Ethics approval

All participants received an invitation letter by regular mail about two weeks prior to receiving the questionnaire, explaining the purpose of the study, that participation was voluntary, and that they could withdraw from the study at any point in time, even after submitting the questionnaire. This information was repeated on the questionnaire itself, and also that consent was presumed if the participant returned a completed questionnaire. This procedure was approved by the Regional Ethical Review Board at Lund University, Sweden (Decision numbers: 1999-99; 2005-471 and 2010-392).

Acknowledgments

Preliminary results from this study were presented by Per-Olof Östergren at the 12th European Public Health Conference in Stockholm, 1-4 November 2017. The authors would like to thank Elizabeth Cantor-Graae for language consulting.

For peer review only

References

1 Alleweldt F, Kara S, Graham R, et. al. (2013). The over-indebtedness of European households: updated mapping of the situation, nature and causes, effects and initiatives for alleviating its impact – Part 1: Synthesis of findings. Retrieved from https://ec.europa.eu/info/sites/default/files/final-report-on-over-indebtedness-of-european-households-synthesis-of-findings_december2013_en.pdf. Date of access 28 January 2022.

2 OECD. (2017). Household debt (indicator) doi: 10.1787/f03b6469-en. Date of access 19 October 2017.

3 Roos J. Konsumtionsrapporten 2014. In. Edited by Archive GUPE: Centrum för konsumtionsvetenskap, Göteborg University; 2014: 54. Retrieved from: http://hdl.handle.net/2077/37778. Date of access 28 January 2022.

4 SOU 2013:72. Ut ur skuldfällan. Betänkande av 2012 års skuldsaneringsutredning. In.: Government offices of Sweden; 2013. Retrieved from: https://data.riksdagen.se/fil/A237B785-C16A-4E50-B6E1-C8CE2AD22442. Date of access 28 January 2022.

5 Simovart MA, Saare K, Sein K. Protection of Consumer Rights in SMS Loan Agreements', *European Review of Private Law* 2010;1:129-42. Retrieved from: https://kluwerlawonline.com/journalarticle/European+Review+of+Private+Law/18.1/ERPL2010006. Date of access 28 January 2022.

6 Ahlström R. Bilaga 1: Överskuldsättningens kostnader. In: *Riksrevisionen granskar.* Riksrevisionen; 2015. Retrieved from: https://www.riksrevisionen.se/download/18.78ae827d1605526e94b2db65/1518435497252/Bilaga1.pdf. Date of access 28 January 2022.

For peer review only - http://bmjopen.bmj.com/site/about/guidelines.xhtml

7 Oyesanya M, Lopez-Morinigo J, Dutta R. Systematic review of suicide in economic recession. *World J Psychiatry* 2015;5:243-54.

8 Frasquilho D, Matos MG, Salonna F, et al. Mental health outcomes in times of economic recession: a systematic literature review. *BMC Public Health* 2016;16:115.

9 Ferreira MB, de Almeida F, Soro JC, et al. On the Relation Between Over-Indebtedness and Well-Being: An Analysis of the Mechanisms Influencing Health, Sleep, Life Satisfaction, and Emotional Well-Being. *Front Psychol* 2021;12:591875. https://doi.org/10.3389/fpsyg.2021.591875

10 Warth J, Beckmann N, Puth MT, et al. Association between over-indebtedness and antidepressant use: A cross-sectional analysis. *Plos One* 2020;15:e0236393.

11 Bialowolski P, Weziak-Bialowolska D, VanderWeele TJ. The impact of savings and credit on health and health behaviours: an outcome-wide longitudinal approach. *Int J Public Health* 2019; 64:573-84.

12 Turunen E, Hiilamo H. Health effects of indebtedness: a systematic review. *BMC Public Health* 2014;14:489.

13 Selenko E, Batinic B. Beyond debt. A moderator analysis of the relationship between perceived financial strain and mental health. *Soc Sci Med* 2011;73:1725-32.

14 Sweet E, Nandi A, Adam EK et al. The high price of debt: household financial debt and its impact on mental and physical health. *Soc Sci Med* 2013;91:94-100.

15 Blomgren J, Maunula N, Hiilamo H. Over-indebtedness and chronic disease: a linked register-based study of Finnish men and women during 1995-2010. *Int J Public Health* 2016;61:535-44.

For peer review only

For peer review only - http://bmjopen.bmj.com/site/about/guidelines.xhtml

16 Hagquist CEI. Economic stress and perceived health among adolescents in Sweden. *J Adolesc Health* 1998;22:250-7.

17 Richardson T, Elliott P, Roberts R. The relationship between personal unsecured debt and mental and physical health: a systematic review and meta-analysis. *Clin Psychol Rev* 2013;33:1148-62.

18 Davies W, Montgomerie J, Wallin S. Financial melancholia: Financial health and indebtedness; 2015. Retrieved at: https://research.gold.ac.uk/id/eprint/16269/1/FinancialMelancholiaMentalHealthandIndebtedness.pdf. Date of access 28 January 2022.

19 Levinsson H, Jiborn M. Minuskontot: Ekonomiska villkor för personer med psykisk funktionsnedsättning. In.: Hjärnkoll/Nationell samverkan för Psykisk Hälsa; 2013. Retrieved from: https://lucris.lub.lu.se/ws/portalfiles/portal/6002117/4864878.pdf. Date of access 28 January 2022.

20 Carlsson F, Merlo J, Lindström M, et al. Representativity of a postal public health questionnaire survey in Sweden, with special reference to ethnic differences in participation. *Scand J Public Health* 2006;34:132-9.

21 Canivet C, Nilsson A, Björk J, et al. Assessment of selection bias due to dropouts in the follow-up of the Scania Public Health Cohort. *Scand J Public Health* 2021;49:457-64.

22 Goldberg DP, Gater R, Sartorius N, et al. The validity of two versions of the GHQ in the WHO study of mental illness in general health care. *Psychol Med* 1997;27:191-7.

23 Goldberg DP, Oldehinkel T, Ormel J. Why GHQ threshold varies from one place to another. *Psychol Med* 1998;28:915-21.

For peer review only

For peer review only - http://bmjopen.bmj.com/site/about/guidelines.xhtml

24 Ahnquist J, Wamala SP: Economic hardships in adulthood and mental health in Sweden. The Swedish National Public Health Survey 2009. *BMC Public Health* 2011;11:788. doi: 10.1186/1471-2458-11-788.

25 Canivet C, Bodin T, Emmelin M, et al. Precarious employment is a risk factor for poor mental health in young individuals in Sweden: a cohort study with multiple follow-ups. *BMC Public Health* 2016;16:687. doi: 10.1186/s12889-016-3358-5

26 Canivet C, Aronsson G, Bernhard-Oettel C, et al. The negative effects on mental health of being in a non-desired occupation in an increasingly precarious labour market. *SSM - Population Health* 2017; 3:516-24. https://doi.org/10.1016/j.ssmph.2017.05.009

27 Zou G. A modified poisson regression approach to prospective studies with binary data. *Am J Epidemiol* 2004;159:702-6.

28 Rothman K. Modern epidemiology. Boston: Little, Brown & Co 1986.

29 Diderichsen F, Hallqvist J, Whitehead M. Differential vulnerability and susceptibility: how to make use of recent development in our understanding of mediation and interaction to tackle health inequalities. *Int J Epidemiol* 2019;48:268-74.

30 Rosvall M, Grahn M, Modén B, et al. Hälsoförhållanden i Skåne: Folkhälsoenkät Skåne 2008: Universitetssjukhuset MAS; 2009. Retrieved from: https://utveckling.skane.se/siteassets/publikationer_dokument/folkhalsoenkat_skane_2008.pdf. Date of access 28 January 2022.

31 Nilsson A, Bonander C, Strömberg U, et al. Reweighting a Swedish health questionnaire survey using extensive population register and self-reported data for assessing and improving the validity of longitudinal associations. *PloS One* 2021;16:e0253969.

For peer review only

32 Hedborg A. Överskuldsättning i kreditsamhället? SOU 2013:78. Retrieved from: https://www.regeringen.se/49bb85/contentassets/7d6ed04741ab4b9badd089ed01fd3039/overskuldsattning-i-kreditsamhallet-sou-201378-hela-betankandet. Date of access 28 January 2022.

33 Lundin A, Hallgren M, Theobald H, et al. Validity of the 12-item version of the General Health Questionnaire in detecting depression in the general population. *Public Health* 2016:136:66-74.

34 Lundin A, Åhs J, Åsbring N, et al. Discriminant validity of the 12-item version of the general health questionnaire in a Swedish case-control study. *Nord J Psychiatry* 2017;71:171-9.

For peer review only

Reporting checklist for cohort study.

Based on the STROBE cohort guidelines.

Instructions to authors

Complete this checklist by entering the page numbers from your manuscript where readers will find each of the items listed below.

Your article may not currently address all the items on the checklist. Please modify your text to include the missing information. If you are certain that an item does not apply, please write "n/a" and provide a short explanation.

Upload your completed checklist as an extra file when you submit to a journal.

In your methods section, say that you used the STROBE cohortreporting guidelines, and cite them as:

von Elm E, Altman DG, Egger M, Pocock SJ, Gotzsche PC, Vandenbroucke JP. The Strengthening the Reporting of Observational Studies in Epidemiology (STROBE) Statement: guidelines for reporting observational studies.

		Reporting Item	Page Number
**Title and abstract**			
Title	#1a	Indicate the study's design with a commonly used term in the title or the abstract	3
Abstract	#1b	Provide in the abstract an informative and balanced summary of what was done and what was found	3
**Introduction**			
Background / rationale	#2	Explain the scientific background and rationale for the investigation being reported	5-7
Objectives	#3	State specific objectives, including any prespecified hypotheses	7
**Methods**			
Study design	#4	Present key elements of study design early in the paper	8-9

For peer review only

Setting	#5	Describe the setting, locations, and relevant dates, including periods of recruitment, exposure, follow-up, and data collection	8-9
Eligibility criteria	#6a	Give the eligibility criteria, and the sources and methods of selection of participants. Describe methods of follow-up.	8-9
Eligibility criteria	#6b	For matched studies, give matching criteria and number of exposed and unexposed	n/a
Variables	#7	Clearly define all outcomes, exposures, predictors, potential confounders, and effect modifiers. Give diagnostic criteria, if applicable	9-10
Data sources / measurement	#8	For each variable of interest give sources of data and details of methods of assessment (measurement). Describe comparability of assessment methods if there is more than one group. Give information separately for for exposed and unexposed groups if applicable.	9-10
Bias	#9	Describe any efforts to address potential sources of bias	10-11
Study size	#10	Explain how the study size was arrived at	8-9
Quantitative variables	#11	Explain how quantitative variables were handled in the analyses. If applicable, describe which groupings were chosen, and why	9-10
Statistical methods	#12a	Describe all statistical methods, including those used to control for confounding	

10-11

Statistical methods	#12b	Describe any methods used to examine subgroups and interactions	10-11
Statistical methods	#12c	Explain how missing data were addressed	8-9
Statistical methods	#12d	If applicable, explain how loss to follow-up was addressed	8-9
Statistical methods	#12e	Describe any sensitivity analyses	

n/a

Results

For peer review only

Participants	#13a	Report numbers of individuals at each stage of study—eg numbers potentially eligible, examined for eligibility, confirmed eligible, included in the study, completing follow-up, and analysed. Give information separately for for exposed and unexposed groups if applicable.	11 and table 1
Participants	#13b	Give reasons for non-participation at each stage	8-9
Participants	#13c	Consider use of a flow diagram	
n/a			
Descriptive data	#14a	Give characteristics of study participants (eg demographic, clinical, social) and information on exposures and potential confounders. Give information separately for exposed and unexposed groups if applicable.	11 table 1
Descriptive data	#14b	Indicate number of participants with missing data for each variable of interest	
table 1			
Descriptive data	#14c	Summarise follow-up time (eg, average and total amount)	
8			
Outcome data	#15	Report numbers of outcome events or summary measures over time. Give information separately for exposed and unexposed groups if applicable.	
11-12 table 2			
Main results	#16a	Give unadjusted estimates and, if applicable, confounder-adjusted estimates and their precision (eg, 95% confidence interval). Make clear which confounders were adjusted for and why they were included	11-13 table 2
Main results	#16b	Report category boundaries when continuous variables were categorized	9-10
Main results	#16c	If relevant, consider translating estimates of relative risk into absolute risk for a meaningful time period	
n/a			

For peer review only

Other analyses	#17	Report other analyses done—eg analyses of subgroups and interactions, and sensitivity analyses	12-13 tables 4a-b13
**Discussion**			
Key results	#18	Summarise key results with reference to study objectives	13
Limitations	#19	Discuss limitations of the study, taking into account sources of potential bias or imprecision. Discuss both direction and magnitude of any potential bias.	15-16
Interpretation	#20	Give a cautious overall interpretation considering objectives, limitations, multiplicity of analyses, results from similar studies, and other relevant evidence.	13-15
Generalisability	#21	Discuss the generalisability (external validity) of the study results	13-15, 17
**Other Information**			
Funding	#22	Give the source of funding and the role of the funders for the present study and, if applicable, for the original study on which the present article is based	17

Notes:

- 13a: 11 and table 1
- 15: 11-12 table 2
- 16a: 11-13 table 2
- 17: 12-13 tables 4a-b13 The STROBE checklist is distributed under the terms of the Creative Commons Attribution License CC-BY. This checklist was completed on 05. February 2022 using https://www.goodreports.org/, a tool made by the EQUATOR Network in collaboration with Penelope.ai

For peer review only
